# Accurate estimation of molecular counts from amplicon sequence data with unique molecular identifiers

**DOI:** 10.1093/bioinformatics/btad002

**Published:** 2023-01-05

**Authors:** Xiyu Peng, Karin S Dorman

**Affiliations:** Department of Epidemiology and Biostatistics, Memorial Sloan Kettering Cancer Center, New York, NY 10065, USA; Department of Statistics, Iowa State University, Ames, IA 50011, USA; Department of Genetics, Development and Cell Biology, Iowa State University, Ames, IA 50011, USA; Bioinformatics and Computational Biology Program, Iowa State University, Ames, IA 50011, USA

## Abstract

**Motivation:**

Amplicon sequencing is widely applied to explore heterogeneity and rare variants in genetic populations. Resolving true biological variants and quantifying their abundance is crucial for downstream analyses, but measured abundances are distorted by stochasticity and bias in amplification, plus errors during polymerase chain reaction (PCR) and sequencing. One solution attaches unique molecular identifiers (UMIs) to sample sequences before amplification. Counting UMIs instead of sequences provides unbiased estimates of abundance. While modern methods improve over naïve counting by UMI identity, most do not account for UMI reuse or collision, and they do not adequately model PCR and sequencing errors in the UMIs and sample sequences.

**Results:**

We introduce Deduplication and Abundance estimation with UMIs (DAUMI), a probabilistic framework to detect true biological amplicon sequences and accurately estimate their deduplicated abundance. DAUMI recognizes UMI collision, even on highly similar sequences, and detects and corrects most PCR and sequencing errors in the UMI and sampled sequences. DAUMI performs better on simulated and real data compared to other UMI-aware clustering methods.

**Availability and implementation:**

Source code is available at https://github.com/DormanLab/AmpliCI.

**Supplementary information:**

Supplementary data are available at *Bioinformatics* online.

## 1 Introduction

Amplicon sequencing has been widely applied to explore complex genetic populations, such as viral quasispecies (Bhiman *et al.*, 2015; Caskey *et al.*, 2017; Jabara *et al.*, 2011; Seifert *et al.*, 2016; Zhou *et al.*, 2015), microbial communities (Callahan *et al.*, 2016; Fields *et al.*, 2021; Karst *et al.*, 2021), cancer tumors (Newman *et al.*, 2016; Shu *et al.*, 2017), immune receptor repertoires (Rosati *et al.*, 2017; Shugay *et al.*, 2014; Vander Heiden *et al.*, 2014) and engineered barcodes (Blundell and Levy, 2014; Kebschull and Zador, 2018; McKenna and Gagnon, 2019). These experiments generate high-coverage sequence data for targeted genomic regions, enabling the detection of variants with single-nucleotide differences or low abundance (Callahan *et al.*, 2017; Hathaway *et al.*, 2018). However, to attach sequencing adapters (Rohland and Reich, 2012) and achieve deep coverage, the targets are amplified by polymerase chain reaction (PCR) and sequenced using error-prone high-throughput technologies. Stochasticity, bias in amplification, along with PCR and sequencing errors obscure the true variants and distort abundances (Kebschull and Zador, 2015; Varghese *et al.*, 2010). Bias is especially problematic in low-biomass samples, like single cells, where few template molecules are intensely amplified (Kivioja *et al.*, 2011; Sze and Schloss, 2019). Errors, chimeras and amplification bias accelerate with more PCR cycles (Sze and Schloss, 2019; Varghese *et al.*, 2010), burying real sequences in increasing noise.

PCR bias is solved by removing PCR-duplicated molecules, but duplicate detection is difficult for amplicon data. Unique molecular identifiers (UMIs), attached to sample molecules before amplification (Hug and Schuler, 2003; König *et al.*, 2010) can directly mark duplicates. UMIs can detect ultra-low frequency variants (Kinde *et al.*, 2011; Xu *et al.*, 2019), eliminate amplification bias (Jabara *et al.*, 2011; Kivioja *et al.*, 2011) and provide cleaner data for downstream analysis (Kim *et al.*, 2020; Svensson, 2020; Townes *et al.*, 2019).

The first UMI processing pipelines (Chen *et al.*, 2019; Shugay *et al.*, 2017; Stoler *et al.*, 2016; Vander Heiden *et al.*, 2014) clustered reads by UMI identity and generated a consensus sample sequence per cluster. Unfortunately, UMIs can *collide*, identically marking two or more sampled molecules (Clement *et al.*, 2018) and leading to undercount, loss or misestimation of sampled variants. And UMIs experience both PCR and sequencing errors, leading to the discovery of false (error) variants and inflated counts of sampled molecules (Smith *et al.*, 2017). Modern deduplication tools account for errors in UMIs, and sometimes, UMI collisions (Supplementary Table S1). Calib (Orabi *et al.*, 2019) clusters whole reads, the UMI and sample sequence, by similarity. Use of whole reads can resolve UMI collisions attached to distinct sample sequences, but clustering without accounting for sequence abundance is difficult to calibrate, likely to over-merge similar reads or under-merge multi-error misreads of abundant haplotypes (Amir *et al.*, 2017; Callahan *et al.*, 2016; Peng and Dorman, 2021). Starcode-umi (Zorita *et al.*, 2015) clusters UMIs and sample sequences separately, using distance and cluster abundance thresholds that need careful tuning (Orabi *et al.*, 2019) then reconstructs error-free reads from denoised UMIs and sample sequences. UMI-tools (Smith *et al.*, 2017) clusters UMIs on a network, using abundance and similarity, but it only avoids UMI collision by partitioning UMIs on reference mapping position.

**Fig. 1.**
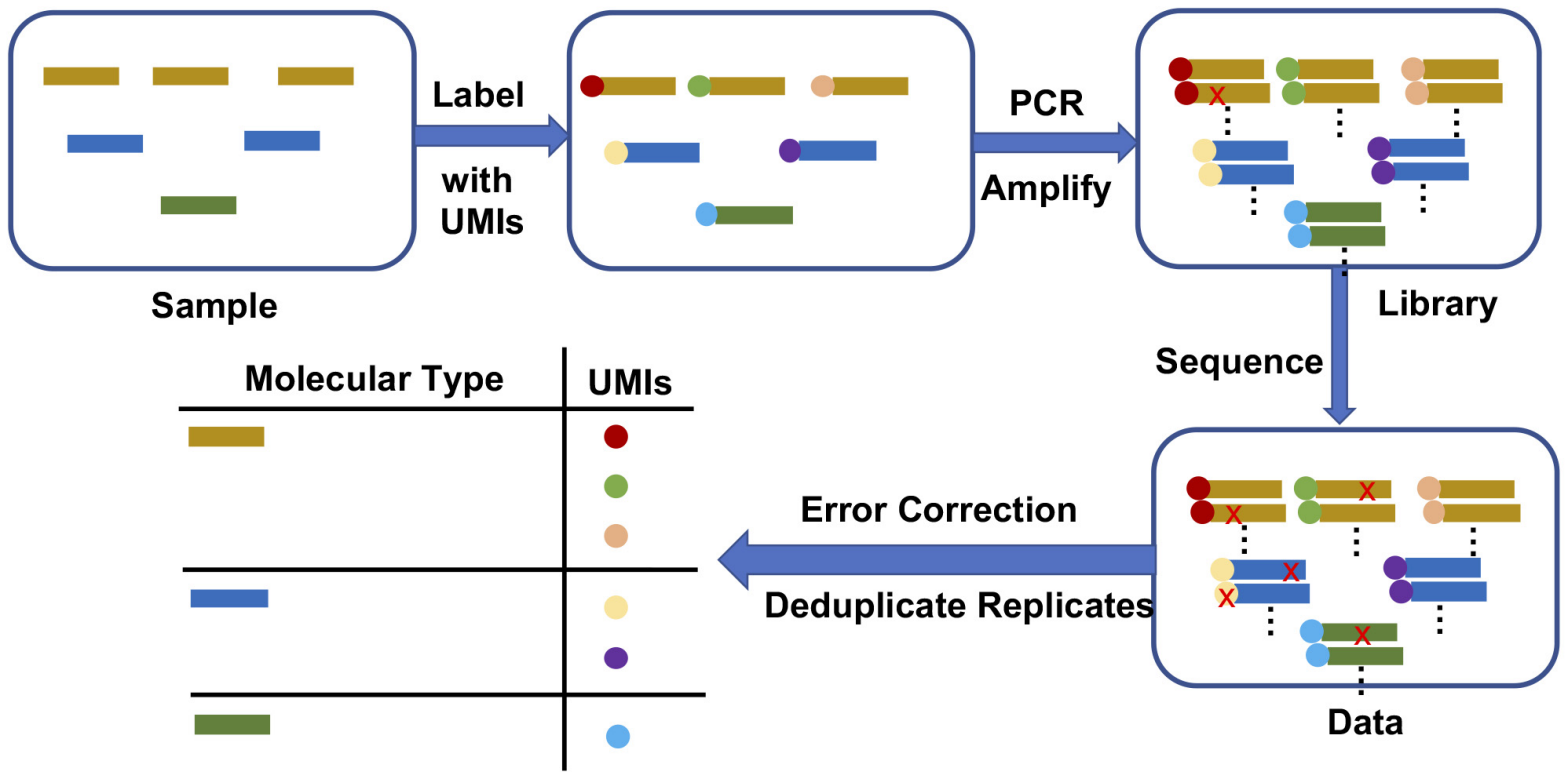
Deduplication and error correction with UMIs. Red crosses are PCR or sequencing errors. Our goal is to resolve the true biological sequences in the sample, as well as their sampled absolute abundance

We introduce DAUMI, Deduplication and Abundance estimation with Unique Molecular Identifiers, a novel probabilistic framework to detect true biological amplicon sequences and accurately estimate their deduplicated abundance (Fig. 1). DAUMI better detects and corrects PCR, sequencing and collision errors on simulation and real data than traditional UMI-aware consensus methods. DAUMI is incorporated in AmpliCI, our amplicon deduplication software, previously only capable of denoising amplicons without UMIs (Peng and Dorman, 2021).

## 2 Materials and methods

We start with a dataset of *n* single-end reads, where the *i*th read contains an observed UMI $b_{i}$ and sample sequence $r_{i}$. A pair $Z_{i}=(Z_{i1},Z_{i2})$ of hidden variables for read *i* encodes the unknown source of UMI sequence $b_{i}$, drawn from a set of *N* true UMIs, $U=\{u_{1},u_{2},\ldots ,u_{N}\}$, and the unknown source of sample sequence $r_{i}$, drawn from a set of *K* true sample sequences (haplotypes), $H=\{h_{1},h_{2},\ldots ,h_{K}\}$.

### 2.1 Model

**Fig. 2.**
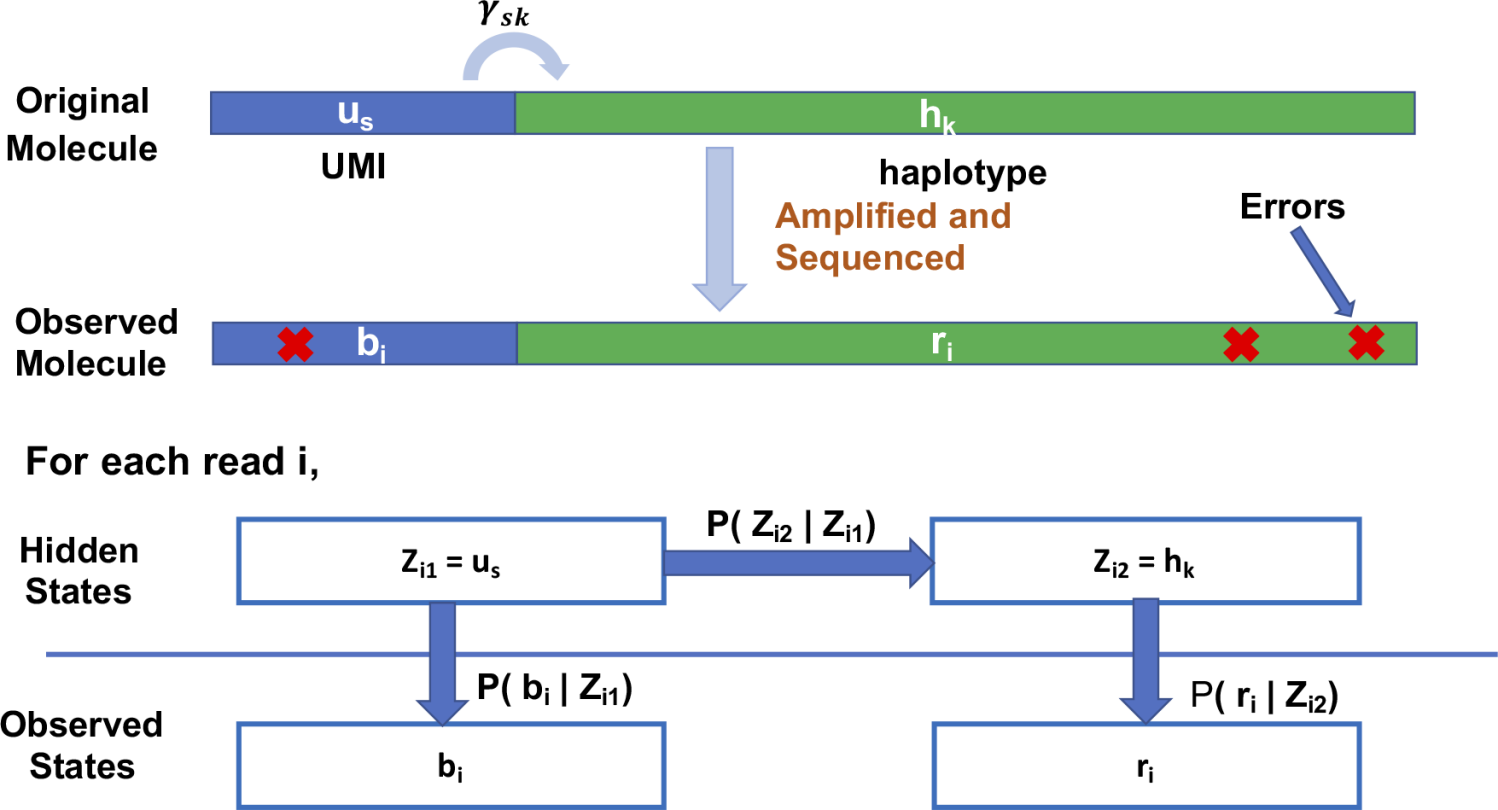
One-step hidden Markov model. A read has two hidden states, true UMI $u_{s}$ attached to haplotype $h_{k}$ by transition matrix $\Gamma =\{\gamma_{sk}\}$, and two observed noisy emission states, UMI $b_{i}$ and sample sequence $r_{i}$

Each read $\left(b_{i},r_{i}\right)$ is assumed to be an independent realization of a one-step Hidden markov model (Fig. 2). True UMI $u_{s}$ is chosen with mixing proportion *η_s_*, a value determined by stochastic amplification, UMI collision and accidental inclusion of low-frequency error UMIs in U. Next, haplotype $h_{k}$ is attached to $u_{s}$ with probability *γ_sk_*. Finally, the observed barcode $b_{i}$ and sample read $r_{i}$ are sequenced from $u_{s}$ and $h_{k}$ with possible errors. The set of all observed barcodes $B=(b_{1},b_{2},\ldots ,b_{n})$ and sample reads $R=(r_{1},r_{2},\ldots ,r_{n})$ has observed log likelihood l(θ|R,B)

(1) $$\sum_{i=1}^{n}\ln[\sum_{h_{k}\in H,u_{s}\in U}\eta_{s}\mathrm{Pr}(b_{i}|Z_{i1}=u_{s})\gamma_{sk}\mathrm{Pr}(r_{i}|Z_{i2}=h_{k})],$$

where θ={η,Γ,H,U}, with $\eta =(\eta_{1},\eta_{2},\ldots ,\eta_{N})$ and $\Gamma ={\left\{\gamma_{sk}\right\}}_{N\times K}$, are model parameters.

We expect $\gamma_{sk}=1_{\left\{k=k_{s}\right\}}$, i.e. only one haplotype $h_{k_{s}}$ is marked by UMI $u_{s}$, but recombination during PCR (e.g. chimeras) (Kebschull and Zador, 2015; Potapov and Ong, 2017) and UMI collision may create more than one positive *γ_sk_* per UMI $u_{s}$. To encourage the sparsity in Γ we expect in a well-executed experiment, we maximize the observed *penalized* log likelihood, where penalty (Armagan *et al.*, 2013; Candès *et al.*, 2008),

(2) $$\rho J(\Gamma )=\rho \sum_{s=1}^{N}\sum_{k=1}^{K}\;\text{log}\;(1+\gamma_{sk}/\omega ),$$

for constants ω,ρ>0, is subtracted from (1). As *ω* approaches 0, the penalty becomes $\ell_{0}$-like, such that estimated transitions ${\hat{\gamma }}_{sk}\approx 0$ when the expected amplified count of molecule $\left(u_{s},h_{k}\right)$ drops below threshold *ρ*. UMI collision is implied when expected counts of $\left(u_{s},h_{k_{1}}\right)$ and $\left(u_{s},h_{k_{2}}\right)$ exceed *ρ*. Because expected counts consider sequence similarity, error rates and abundance, DAUMI can identify UMI collision even on highly similar sampled molecules (Supplementary Fig. S1).

For emission probabilities $\mathrm{Pr}(b_{i}|Z_{i1})$ and $\mathrm{Pr}(r_{i}|Z_{i2})$, we use an existing sequencing error model (Peng and Dorman, 2021). First, we align the read and the candidate UMI and haplotype $\left(Z_{i1},Z_{i2}\right)$. The emission of observed read ***X*** ($b_{i}$ or $r_{i}$ with indels) from true sequence ***Y*** ($Z_{i1}$ or $Z_{i2}$ with indels) is

(3) $$\mathrm{Pr}(X|Y)=\mathrm{Pr}(d|Y)\prod_{j=1}^{l}\mathrm{Pr}(X_{j}|Y_{j};q_{j}),$$

where *l* is the alignment length, *d* is the number of observed insertion/deletion (indel) events, and $\mathrm{Pr}(X_{j}|Y_{j};q_{j})$ is the probability (1 for indels) of generating *X_j_* from *Y_j_* with quality score *q_j_* at aligned position *j*. Indels follow a truncated Poisson(δl) with indel error rate *δ* per position per read, but we assume *d *=* *0 in UMIs since indel errors are rare in these short, typically high quality parts of the reads.

The goal of DAUMI is to estimate true haplotypes and abundances. We choose candidate haplotypes H, UMIs U and parameters of (3) (see Section 2.2.1). Then, we maximize the penalized log likelihood to obtain estimates $\hat{\eta }$ and $\hat{\Gamma }$ using an expectation–maximization (EM) algorithm (details in Supplementary Section S1; demonstrated accuracy in Supplementary Fig. S8). H and U are fixed, but we can discard UMIs with expected abundance $n{\hat{\eta }}_{s}<1$ and the penalty may eliminate false haplotypes. Finally, the deduplicated abundance of haplotype $h_{k}$ is $\sum_{s=1}^{N}1_{\left\{n{\hat{\eta }}_{s}\geq 1\right\}}1_{\left\{{\hat{\gamma }}_{sk}>0\right\}}$.

### 2.2 Implementation

DAUMI is implemented in C. We describe EM algorithm initialization, approximations used for speed, and selection of penalty parameters.

#### 2.2.1 Initialization

To include all true UMIs and haplotypes in candidate pools U and H, while excluding most error UMIs and haplotypes, we extend UMI-unaware AmpliCI (Peng and Dorman, 2021). We set U to the ‘denoised’ UMIs (option ––umi) assuming quality scores provide PHRED error probabilities (Ewing and Green, 1998). Quality scores are generally accurate (Zanini *et al.*, 2017), but they slightly underestimate error rates (Schirmer *et al.*, 2016; Shugay *et al.*, 2017), suggesting this approach will liberally identify UMIs. To populate H, AmpliCI is applied to full reads (UMI length set via –-trim_umi_length), using an error profile estimated from reads clustered by the above-denoised UMIs, further split by a UMI-tools-like algorithm (Smith *et al.*, 2017). Specifically for error profile estimation, if any sample sequence in a UMI cluster has over half the abundance of the most abundant sample sequence, we partition the cluster as a possible UMI collision. The two sequences are promoted to new cluster centers, and each read in the original cluster is assigned to the closer center, by Hamming distance, or the more abundant center if equidistant. To ensure cluster centers are error free, we drop clusters where the center sequence is not sufficiently replicated (default, observed abundance < 2), then assume all differences between cluster members and cluster centers are errors. The fitted error profile is plugged into (3) and the unique, ‘denoised’ sequences, without UMI tags, form H. Finally with U and H given, we initialize mixing proportions η to relative observed abundances of UMIs and transition matrix Γ to observed transition counts, normalizing row sums to one.

#### 2.2.2 Approximations

For large datasets, we reduce computation and memory expended on unlikely UMI-haplotype combinations. Since Illumina errors are <0.1% per nucleotide (Stoler and Nekrutenko, 2021), we assume each true molecule $\left(u_{s},h_{k}\right)$ has been observed without error at least once and otherwise force $\gamma_{sk}\equiv 0$. Most surviving combinations have exceptionally small posterior probabilities *e_isk_* (defined in Supplementary Section S1). In the E step, we only keep the top *T* (default 10) most likely candidates for each read *i*, resetting

$$e_{\mathrm{isk}}=1_{\left\{e_{\mathrm{isk}}\geq e_{\mathrm{isk},(NK-T+1)}\right\}}\frac{e_{\mathrm{isk}}}{\sum_{t=1}^{T}e_{\mathrm{isk},(NK-t+1)}},$$

where $e_{\mathrm{isk},(NK-T+1)}$ is the *T*th largest value among *NK* entries. With these approximations, estimate ${\hat{\eta }}_{s}=0$ is possible, in which case we assume $u_{s}$ is an error UMI and exclude the *s*th row of Γ. Increasing *T* to 30 had no effect on estimation in simulation (data not shown). DAUMI computational complexity depends on the number of UMIs |U|, haplotypes |H| and EM iterations (see Supplementary Table S3). Additional improvements, especially in the E step, are likely possible.

#### 2.2.3 Selection of the penalty parameters

Fixing $\omega =10^{-20}$ for an $\ell_{0}$ penalty (Yin, 2016), *ρ* is chosen to separate true from error UMIs. To automate *ρ* selection, we model the observed UMI abundance distribution. Specifically, we modify a Galton–Watson branching process (Stolovitzky and Cecchi, 1996) to account for amplification and sequencing error. We fit the model by minimizing the Kolmogorov–Smirnov statistic (Stephens, 1974) applied to the observed UMI abundances right-truncated for long tails caused by UMI collision or other artifacts. Selecting *ρ* as the fifth percentile of the fitted distribution worked well for all datasets, but we manually selected *ρ *= 20 for HIV V3 based on visual assessment. Details and limitations of this procedure are provided in Supplementary Material Section S2. Supplementary Table S4 shows fitted models and selected *ρ* for all datasets.

### 2.3 Data simulation

We conducted a brief simulation (pipeline in Supplementary Fig. S2 and settings, including PCR efficiencies and cycle numbers, in Supplementary Table S2). The relative abundance π of *K *=* *25 haplotypes were obtained from power law distribution $f(x)\propto x^{-0.5}1_{\left\{0\leq x\leq 1\right\}}$. Then, *N *=* *400 molecules were sampled from a Multinomial(N,π) distribution and randomly attached to *N* 9 bp UMIs. For datasets with UMI collision, we resampled the *N* distinct UMIs *with replacement* before attachment. The 400 true UMIs and 25 true haplotypes were randomly selected from UMIs and haplotypes found in an HIV dataset (Bhiman *et al.*, 2015, SRR2241783) after AmpliCI denoising (Peng and Dorman, 2021). Next, molecules were PCR amplified with error probability per base, per cycle from Quince *et al.* (2011) ($10^{-4}$–$10^{-6}$, depending on substitution). ART Illumina (Huang *et al.*, 2012) simulated single-end reads with fixed length 250 bp, using error profile MSv1 to mimic Miseq reads. Indel rates were set to $2\times 10^{-5}$ per position.

### 2.4 Running competing methods

We ran Calib (Orabi *et al.*, 2019), UMI-tools (Smith *et al.*, 2017), Starcode-umi (Zorita *et al.*, 2015) and a ‘Naïve method’ that clusters reads by UMI identity (Clement *et al.*, 2018; Vander Heiden *et al.*, 2014) with default parameter settings, except as discussed here. Since Calib (v0.3.4) requires paired-end reads, we passed in UMI-tagged reads as forward reads and UMI-detagged reads as reverse reads (a hack suggested by the author). We disabled sequence trimming (–-seq-trim 0) in Starcode-umi (v1.3) to ensure a fair comparison with other algorithms, and set maximum edit distance one (–-umi-d 1), a common setting for UMI clustering. For UMI-tools, we used the core algorithm from its Application Programming Interface, which is stable across versions, to cluster UMIs. For the Naïve method, sequences were clustered by UMI identity, discarding singleton UMIs and attached sample sequences as likely errors. For Calib, UMI-tools and the Naïve method, we took the majority rule consensus sequence for the true UMI and haplotype (first seen of {A, T, C, G} when tied) per cluster. For Starcode-umi, we used the canonical UMIs and sample sequences estimated during clustering. Supplementary Table S6 verifies chosen parameters were optimal or near-optimal for all methods (details in Supplementary Section S3).

## 3 Results

We compared DAUMI to four UMI clustering methods, Calib (Orabi *et al.*, 2019), UMI-tools (Smith *et al.*, 2017), Starcode-umi (Zorita *et al.*, 2015) and the Naïve method, on both simulated and real datasets. For comparison to UMI-unaware AmpliCI, see Supplementary Figure S7.

### 3.1 Benchmarking on simulated data

To compare methods, we simulated four datasets (Supplementary Table S2). Of 25 true haplotypes, only 23 were sampled in Simulation 2 and 22 in the others. Calib ran on subsets of Simulation 2 (50%), 3 (45%) and 4 (50%), since it failed on the whole dataset (true abundances adjusted accordingly).

**Fig. 3.**
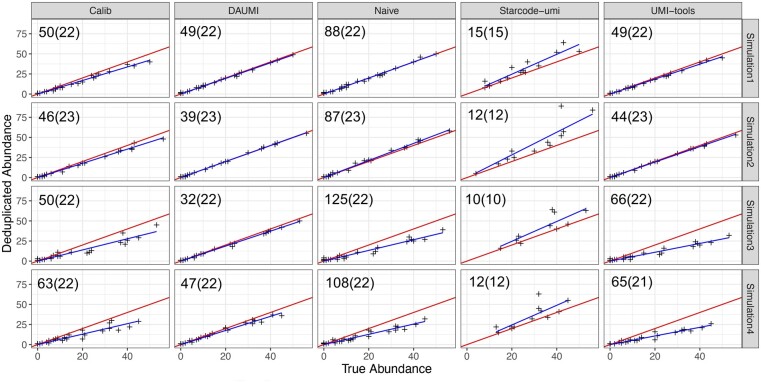
Abundance estimation on simulated data *without* (Simulations 1 and 2) or *with* (Simulations 3 and 4) UMI collision. Blue is fitted (*y *=* bx*) and red is expected (*y *=* x*) line. Number of estimated (true) haplotypes at upper left of each scatter plot

DAUMI is superior in all four simulations (Fig. 3, estimates and goodness-of-fit in Supplementary Table S5). *Without* UMI collision, DAUMI, Calib, UMI-tools and Naïve identify all true haplotypes, but DAUMI introduces fewer false positives (Fig. 3, Simulations 1 and 2). Starcode-umi finds less true haplotypes and is the only method to overestimate abundance; its estimates also contain more noise. DAUMI excels on data *with* UMI collision (Fig. 3, Simulations 3 and 4). UMI-tools and Naïve, which cannot detect collision, underestimate haplotype abundance, but Calib also underestimates abundance, possibly by merging UMI clusters with similar tagged reads.

### 3.2 Application to HIV amplicon data

We analyzed reverse reads with attached UMIs from two HIV *env* amplicon datasets, V1V2 (Bhiman *et al.*, 2015, SRR2241783) and V3 (Caskey *et al.*, 2017, SRR5105420). For V1V2, UMIs were extracted from the reads using a custom method (Supplementary Section S5). For V3, UMIs were extracted from the read starts, dropping the following primer sequences. Reads with ambiguous nucleotides (1%) were discarded, and remaining reads were trimmed to the same length (details in Supplementary Table S8).

To assess performance when true haplotypes and abundances are unknown, we evaluated method consistency across a random bipartition (repeated five times). We report for each subset (Table 1) average number of haplotypes found and two similarity metrics: Jaccard Index measures agreement in found haplotypes (Jaccard, 1912); Ruzicka Similarity (Deza and Deza, 2006) is further weighted by estimated abundances. Methods are more consistent across subsets for V1V2 (Supplementary Fig. S11) than V3 (Supplementary Fig. S9).

**Table 1.** Agreement across random partition of HIV V1V2 and V3 datasets

		Deduplicated abundance ≥ 1							Deduplicated abundance ≥ 2						
Dataset	Methods	Hap1	Hap2	SD	Jaccard		Ruzicka		Hap1	Hap2	SD	Jaccard		Ruzicka	
V1V2	Calib	808	797	(21)	0.08	(0.00)	0.23	(0.00)	43	43	(4)	0.67	(0.03)	0.82	(0.02)
	DAUMI	90	90	(5)	**0.72**	(0.03)	**0.84**	(0.02)	37	37	(2)	**0.77**	(0.05)	0.87	(0.02)
	Naïve	293	303	(9)	0.29	(0.01)	0.57	(0.01)	43	43	(3)	0.75	(0.03)	**0.88**	(0.03)
	Starcode-umi	2304	2311	(48)	0.00	(0.00)	0.19	(0.00)	15	13	(1)	0.73	(0.05)	0.80	(0.02)
	UMI-tools	542	538	(16)	0.14	(0.00)	0.36	(0.00)	42	42	(3)	**0.77**	(0.04)	0.87	(0.01)
V3	Calib	5048	5021	(57)	0.06	(0.00)	0.11	(0.00)	64	66	(4)	0.55	(0.04)	0.71	(0.02)
	DAUMI	180	171	(7)	**0.39**	(0.01)	**0.72**	(0.01)	68	72	(4)	**0.72**	(0.03)	**0.83**	(0.01)
	Naïve	1561	1543	(10)	0.06	(0.00)	0.22	(0.00)	91	84	(4)	0.64	(0.04)	0.74	(0.02)
	Starcode-umi	15077	15097	(79)	0.02	(0.00)	0.09	(0.00)	96	103	(5)	0.08	(0.01)	0.77	(0.02)
	UMI-tools	3718	3724	(36)	0.06	(0.00)	0.14	(0.00)	72	74	(3)	0.64	(0.03)	0.75	(0.01)

*Note*: The mean and standard deviation (SD in parentheses) of five replicates of: Hap1 and Hap2: no. inferred haplotypes in each subset (rounded to integer); Jaccard: Jaccard Index of inferred haplotype sets; Ruzicka: Ruzicka Similarity, abundance-weighted form of Jaccard Index. For DAUMI, *ρ* = 10 for V3, *ρ* = 7 for V1V2, both subsets. Best performance is bolded per dataset.

**Fig. 4.**
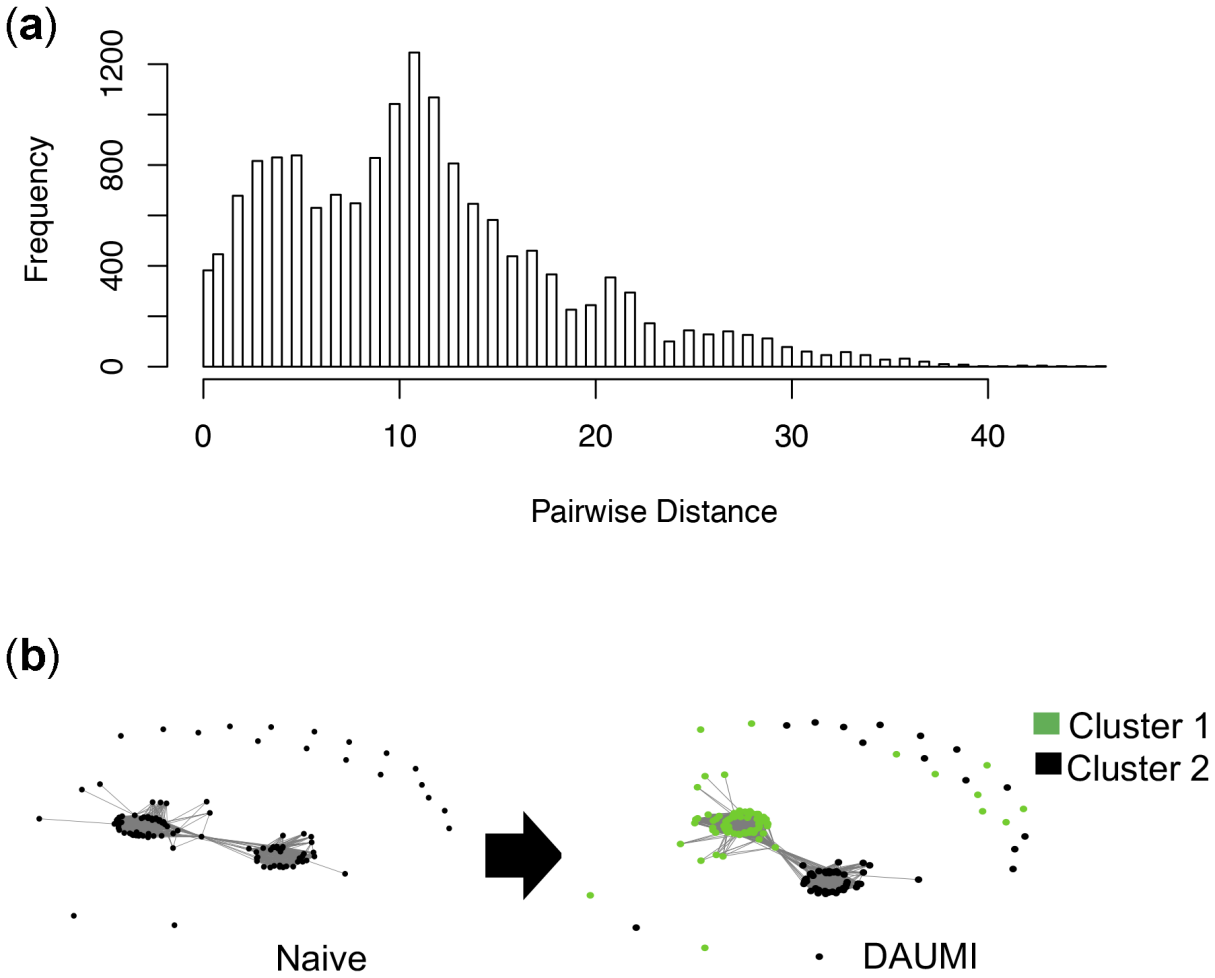
UMI collision in V1V2 data resolved by DAUMI. (**a**) Distribution of pairwise distances between unique sequences labeled with UMI GTGTCGGTA, indicating at least two clusters linked this UMI. (**b**) DAUMI finds two clusters (green, left, and black, right). Network nodes are unique sample sequences; edges link sequences within edit distance six

Overall, DAUMI achieves the highest agreement in recovered haplotypes and estimated abundances on both HIV datasets (Table 1). Not surprisingly, abundant haplotypes are easier to detect, but DAUMI can also reliably find low-abundance haplotypes. Only UMI-tools and Naïve are competitive and only on the subset of haplotypes attached to at least two UMIs in V1V2. Though UMI collision is rare in these data, DAUMI and Starcode-umi detect a collision on two haplotypes with nine mismatches (Fig. 4). Within this UMI cluster (126 sequences), DAUMI infers two haplotype sequences with high observed abundance (15 and 7) that also dominate other UMI clusters. Starcode-umi finds one of these haplotypes but chooses for the other a sequence never seen in the reads. DAUMI substantially outperforms all competing methods on V3, probably because of an elevated collision rate. Supplementary Figures S5 and S6 show a long right tail despite low PCR amplification, and Supplementary Figure S10 finds excess nucleotide T, suggesting degeneracy in the random UMIs.

### 3.3 Application to single-cell data

We further compared methods on a SMART-Seq3 scRNA-seq benchmark dataset (Ziegenhain *et al.*, 2022, E-MTAB-10372). These data include a molecular spike carrying an 18-bp internal UMI. Molecular counting by the 8 bp SMART-Seq3 UMI can be calibrated by comparing to counts of the 18-bp spike UMIs. DAUMI is not intended for general scRNA-seq data, but it can be applied to this molecular spike data and targeted single-cell data (Pokhilko *et al.*, 2021; Woyke and Jarett, 2015). We demultiplexed using pheniqs (Galanti *et al.*, 2021) and retained only 5′ reads of the molecular spike. These reads contain a 57-bp molecular spike sequence, including the 18-bp spike UMI and surrounding fixed sequence, which we treat as the sampled amplicon. Only reads containing the spike at the expected location and no ambiguous nucleotides are kept.

**Fig. 5.**
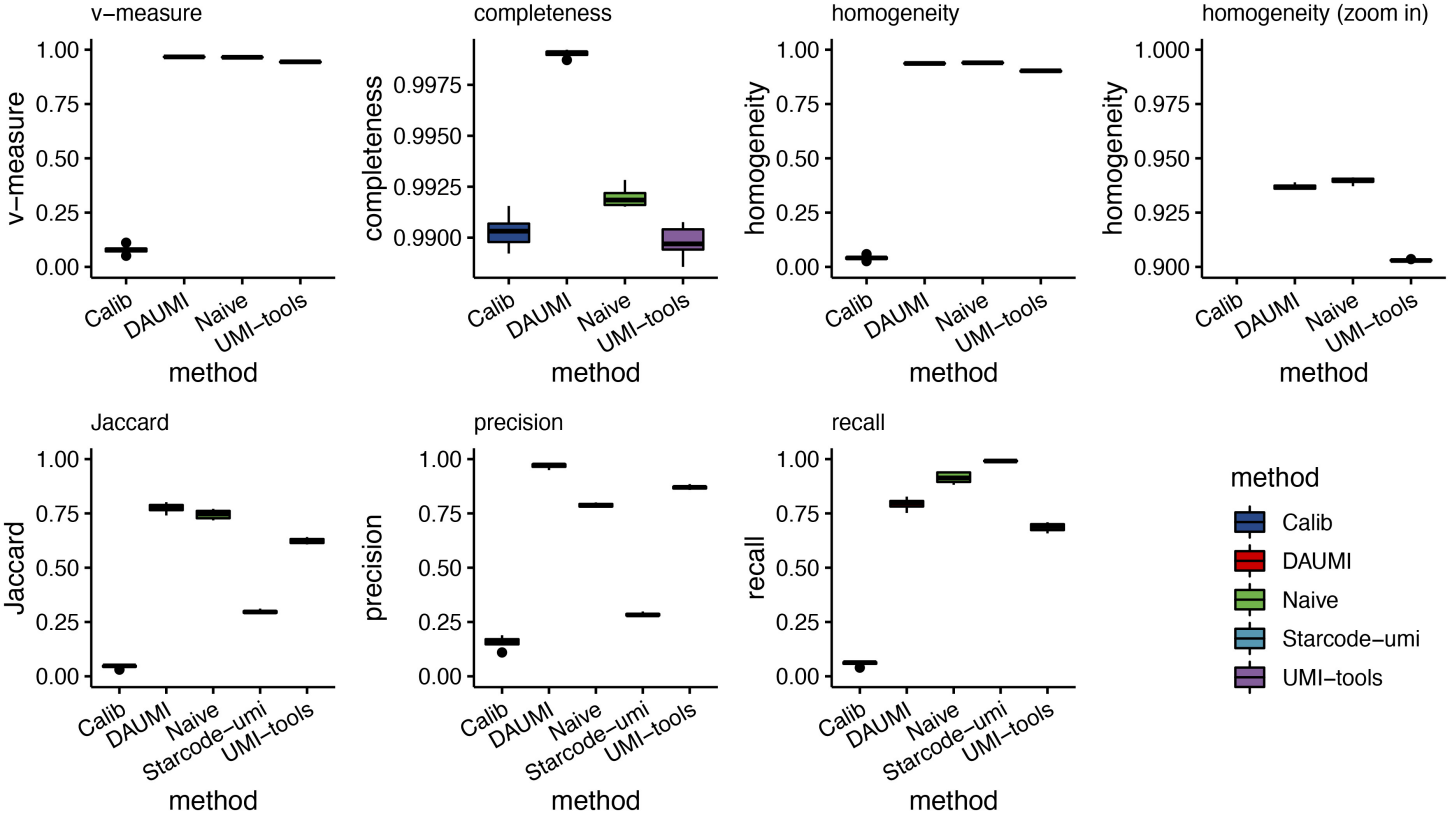
Single-cell molecular spike data. Jaccard index, precision and recall were computed by comparing the resolved haplotypes with the DADA2-estimated gold standard for each cell. Cluster homogeneity and completeness were computed by treating the 18 bp raw spike UMIs as the true class label (Starcode-umi does not provide read assignments). For DAUMI, *ρ *= 9 was auto-selected for all 41 cells

For 41 cells, we denoised the 18-bp spike UMIs using DADA2 (Callahan *et al.*, 2016) with singletons (DETECT_SINGLETONS = TRUE) to produce gold-standard haplotypes. When comparing resolved haplotypes to the gold standard, DAUMI had the highest precision but lower recall than Naïve and Starcode-umi (Fig. 5), probably because the initialization of the UMI set U is conservative on this very clean data (Supplementary Table S11). When evaluating clusters, Naïve produced slightly more homogeneous clusters than DAUMI, but DAUMI had far superior completeness (Dorman *et al.*, 2021). Naïve oversplits clusters, spreading spike UMIs across multiple clusters, leading to overestimated UMI counts (and low completeness).

## 4 Discussion

DAUMI is a novel probabilistic framework to correct errors and deduplicate reads for accurate molecular counts of amplicon sequence data with UMIs. DAUMI correctly resolves the origin of more molecules than competing methods even with UMI collisions. Here, we discuss advantages, limitations and directions for future research.

UMI collision happens, especially when UMIs are short (Clement *et al.*, 2018). Low nucleotide diversity in high-frequency UMIs was reported for single-cell RNA-seq (scRNA-seq) (Petukhov *et al.*, 2018), and we observed similar patterns in the V3 data. Most UMI-based quantification methods for scRNA-seq, e.g. UMI-tools derivative Alevin (Srivastava *et al.*, 2019), map reads to avoid UMI collision, but mapping cannot resolve amplicons. Even in scRNA-seq, mapping may under-collapse clusters (Srivastava *et al.*, 2019), since reads of the same molecule can map to slightly different genome positions (Sena *et al.*, 2018). Calib and Starcode-umi can detect UMI collision but performed overall worse than UMI-tools and DAUMI.

DAUMI and all methods compared here cannot detect errors introduced before UMI tagging, e.g. during cDNA synthesis. Downstream methods may detect such errors, like UMI-based variant callers (Shugay *et al.*, 2017; Xu *et al.*, 2019), which compare variation to background error rates after UMI clustering. Methods also struggle to detect early-cycle PCR errors, which are highly amplified and difficult to distinguish from true variation. If such errors occur in the UMI, methods may overestimate abundance by treating both UMIs as valid. *Post hoc* detection is possible by checking for similar UMIs attached to identical haplotypes. More likely, errors strike the sampled molecule. Other methods vary in their resolution of such cases, but DAUMI will treat the result as a UMI collision and overestimate the abundance of a false haplotype. If good experimental design has eliminated UMI collision, DAUMI abundance estimation can assign each UMI to one and only one haplotype, namely $\text{arg}{\text{max}}_{k}{\hat{\gamma }}_{sk}$ (with option –-ncollision), but when collision and early-cycle PCR error coexist, explicit modeling of the PCR process may be necessary.

DAUMI is moderately affected by penalty parameter *ρ*, a molecular count distinguishing error and true molecules. With UMI collisions, high *ρ* may merge similar haplotypes, and regardless of UMI collision, low *ρ* may over-split (overcount in UMI; false positive in haplotype) PCR errors. Automated selection of *ρ* worked well for simulated, V1V2, and single-cell data. For V3, with low UMI replication, we manually overrode the auto-selected *ρ* = 1 with *ρ* = 20, but both choices worked well (Supplementary Table S9). We also demonstrated that DAUMI has superior performance for a range of reasonable *ρ* (Supplementary Table S6). It may be possible to use cross-validation in large datasets or control variants to calibrate *ρ* or adaptively select distinct *ρ_sk_* for each UMI/haplotype combination, perhaps as a simple function of $u_{s},h_{k}$ abundance or the proximity and abundance of other observed molecules. Such attempts are increasingly complex approximations of an explicit PCR model.

DAUMI is affected by the candidate UMI U and haplotype H sets. If candidates are known a priori, DAUMI works very well (Supplementary Section S4), but prior knowledge of H is rare. We used UMI-unaware AmpliCI (Peng and Dorman, 2021) to populate U and H. This method assumes each true sample sequence is observed with the same UMI at least twice. Singleton discard is a blunt way to increase agreement across subsets, as shown for Calib, UMI-tools and Starcode-umi, which do not discard singletons by default (Table 1 versus Supplementary Table S10). The resulting UMI counts are biased downward, which can be corrected (Pflug and von Haeseler, 2018), but the discarded haplotypes cannot be recovered. In low-complexity samples with low amplification or high downsampling, multiple singleton or low-frequency UMIs may be linked to the same sampled molecule. DAUMI’s poorer performance on V1V2 when restricting to haplotypes with deduplicated abundance ≥2 (Supplementary Table S10) is due to the discard of barely amplified UMI-tagged haplotypes. AmpliCI sensitivity can be increased (see options –-diagnostic, –-useAIC, –-abundance), but the defaults used here are set to work on varied datasets and without posthoc filtering.

DAUMI is easily extended. Overpopulation of UMI set U could be ameliorated by penalizing the relative abundances *η_s_* of $u_{s}\in U$. A hierarchical model on *η_s_* could detect early PCR errors or other sources of variation in UMI abundance, such as biased nucleotide usage. Transition probabilities in Γ that link UMI $u_{s}$ to haplotype $h_{k}$ could model transcript selection followed by random fragmentation, as utilized in some scRNA-seq protocols (Zilionis *et al.*, 2017). Finally, the DAUMI framework is not limited to Illumina sequence data and could, with appropriate error model and good initialization, be applied to long-read technology.

## Supplementary Material

btad002_Supplementary_Data

## Data Availability

Code and links to existing data resources used to in this manuscript can be found in the public repository for the software: https://github.com/DormanLab/AmpliCI.
