# Supplementary material for: Accurate estimation of molecular counts from amplicon sequence data with unique molecular identifiers
Source: Bioinformatics. 2023 Jan 5;39(1):btad002. doi: 10.1093/bioinformatics/btad002 (PMC9891248; doi:10.1093/bioinformatics/btad002)
Supplement: btad002_Supplementary_Data [file btad002_supplementary_data.pdf]

Supplementary Material for "Accurate Estimation of Molecular Counts from Amplicon Sequence Data with Unique Molecular Identifiers"

Xiyu Peng[1], Karin S. Dorman[2,3,4*]

[1]*Department of Epidemiology and Biostatistics, Memorial Sloan Kettering Cancer Center*

[2]*Department of Statistics, Iowa State University*

[3]*Department of Genetics, Development and Cell Biology, Iowa State University*

[4]*Bioinformatics and Computational Biology Program, Iowa State University*

January 5, 2023

Contents

S1 EM Algorithm **1**

S2 Automated Selection of Penalty Parameter ρ **4**

S3 Tuning Run Parameters of Other Methods **7**

S4 Abundance Estimation for Known Variants **7**

S5 Extracting UMIs from V1V2 Dataset **8**

S6 Supplementary Figures and Tables **12**

S1 EM Algorithm

We use an EM algorithm (see listing 1) to estimate UMI proportions $\boldsymbol{\eta}$ and the transition parameters $\boldsymbol{\Gamma}$ that link UMIs to sample sequences. Conditional on the set of N UMI candidates in $\mathcal{U}$ and K haplotype candidates in $\mathcal{H}$, the complete data penalized log likelihood function for both the observed data, errored

Algorithm 1: EM algorithm

1. Initialize $\boldsymbol{\eta}, \boldsymbol{\Gamma}$, candidate haplotype set $\mathcal{H}$, candidate UMI set $\mathcal{U}$ (see §2.2.1).
2. E step: Given the current parameters $\boldsymbol{\eta}$ and $\boldsymbol{\Gamma}$, compute:
   (a) the posterior probability e_{isk} for all reads i, selecting T UMIs $\boldsymbol{u}_s \in \mathcal{U}$ and haplotypes $\boldsymbol{h}_k \in \mathcal{H}$ (see §2.2.2) using Eq. (S3), and
   (b) observed data penalized log likelihood $l(\boldsymbol{\eta}, \boldsymbol{\Gamma} \mid \mathcal{R}, \mathcal{B}) - \rho \mathcal{J}(\boldsymbol{\Gamma})$.
3. M step: Given e_{isk} from the E step, update:
   (a) $\boldsymbol{\eta}$: $\eta_s^{(t+1)} = \frac{1}{n}\sum_{i=1}^{n}\sum_{k=1}^{K} e_{isk}$, and
   (b) $\boldsymbol{\Gamma}$ by maximizing (S5) using the Newton-Raphson algorithm (this section).
4. Iterate the E step and the M step until convergence.

reads $\mathcal{R}$ and barcodes $\mathcal{B}$, and unobserved hidden variables $\boldsymbol{W}$ is

$$l(\boldsymbol{\eta}, \boldsymbol{\Gamma} \mid \mathcal{R}, \mathcal{B}, \boldsymbol{W}) = \sum_{i=1}^{n}\sum_{s=1}^{N}\sum_{k=1}^{K} \mathbb{1}_{\{Z_{i1}=\boldsymbol{u}_s, Z_{i2}=\boldsymbol{h}_k\}} \ln\big[\eta_s \Pr(\boldsymbol{b}_i \mid \boldsymbol{u}_s)\gamma_{sk} \Pr(\boldsymbol{r}_i \mid \boldsymbol{h}_k)\big] - \rho\mathcal{J}(\boldsymbol{\Gamma}). \tag{S1}$$

Define the conditional expectation of the hidden data $\boldsymbol{Z}_i = (Z_{i1}, Z_{i2})$ given the observed data $(\boldsymbol{b}_i, \boldsymbol{r}_i)$ and computed with the current parameter estimates $\boldsymbol{\eta}^{(t)}$ and $\boldsymbol{\Gamma}^{(t)}$,

$$e_{isk}^{(t)} = \Pr(Z_{i1} = \boldsymbol{u}_s, Z_{i2} = \boldsymbol{h}_k \mid \boldsymbol{r}_i, \boldsymbol{b}_i; \boldsymbol{\eta}^{(t)}, \boldsymbol{\Gamma}^{(t)}). \tag{S2}$$

The EM algorithm iterates between an E step, where the expectations $e_{isk}^{(t)}$ are computed, and an M step, where the model parameters are estimated to maximize equation (S1), with $e_{isk}^{(t)}$ substituted for $\mathbb{1}_{\{Z_{i1}=\boldsymbol{u}_s, Z_{i2}=\boldsymbol{h}_k\}}$. Iterations will continue to a local maximum of Eq. (1) when the relative change in observed data log likelihood drops below a threshold (default, 1×10^{-6}). To simplify the notation in the following, we drop the superscripts (t) indicating the tth EM iteration, but each of e_{isk}, ξ_{sk}, λ_s, ϕ_{sk}, some to be defined below, as well as the parameters $\boldsymbol{\eta}, \boldsymbol{\Gamma}$ and their updates $\hat{\boldsymbol{\eta}}, \hat{\boldsymbol{\Gamma}}$, will depend on the iteration t.

E step:

For the ith read, e_{isk} is, by Bayes rule,

$$e_{isk} = \frac{\eta_s \Pr(\boldsymbol{b}_i \mid \boldsymbol{u}_s)\gamma_{sk} \Pr(\boldsymbol{r}_i \mid \boldsymbol{h}_k)}{\sum_{\boldsymbol{u}_s \in \mathcal{U}, \boldsymbol{h}_k \in \mathcal{H}} \eta_s \Pr(\boldsymbol{b}_i \mid \boldsymbol{u}_s)\gamma_{\boldsymbol{u}_s,\boldsymbol{h}_k} \Pr(\boldsymbol{r}_i \mid \boldsymbol{h}_k)}, \tag{S3}$$

where all probabilities are given in the main text and parameters are replaced with their current estimates.

M step:

Parameter $\boldsymbol{\eta}$ can be updated with

$$\hat{\eta}_s = \frac{1}{n} \sum_{i=1}^{n} \sum_{k=1}^{K} e_{isk}, \tag{S4}$$

while the sth row $\boldsymbol{\gamma}_s$ of transition matrix $\boldsymbol{\Gamma}$ can be updated by maximization of

$$\sum_{i=1}^{n} \sum_{k=1}^{K} e_{isk} \log(\gamma_{sk}) - \rho \sum_{k=1}^{K} \log(1 + \gamma_{sk}/\omega) - \lambda_s \left(\sum_{k=1}^{K} \gamma_{sk} - 1 \right), \tag{S5}$$

where λ_s is the Lagrange multiplier to impose the sum constraint for transitions out of $\boldsymbol{u}_s$. In the absence of a penalty ($\rho = 0$), the MLE is

$$\hat{\gamma}_{sk} = \frac{\xi_{sk}}{\sum_{k=1}^{K} \xi_{sk}},$$

where $\xi_{sk} = \sum_{i=1}^{n} e_{isk}$ is the expected number of molecules where UMI $\boldsymbol{u}_s$ is attached to molecule $\boldsymbol{h}_k$. For positive ρ, the maximum penalized-likelihood estimators (MPLEs) of $\boldsymbol{\gamma}_s$ are $\hat{\gamma}_{sk} = 0$ when $\xi_{sk} = 0$ and solutions of the score functions,

$$\frac{\xi_{sk}}{\gamma_{sk}} - \frac{\rho}{\omega + \gamma_{sk}} - \lambda_s = 0,$$

for each k with $\xi_{sk} > 0$. Yin *et al.* (Yin, 2016) prove the solution to these score functions exists and maximizes the objective function when (a) at least one $\xi_{sk} > \rho$ and (b) $|\xi_{sk} - \rho| > \omega$ for all k. Under these conditions, $\hat{\gamma}_{sk}$ is the valid root of

$$\frac{\phi_{sk} \pm \sqrt{\phi_{sk}^2 + 4\omega\lambda_s\xi_{sk}}}{2\lambda_s},$$

where $\phi_{sk} = \xi_{sk} - \omega\lambda_s - \rho$ and λ_s is the root of implicit equation

$$\sum_{k=1}^{K} \hat{\gamma}_{sk} = 1. \tag{S6}$$

In practice, we set ω to a very small value (default: 10^{-20}) for LASSO-like regularization and apply the Newton-Raphson algorithm to find the root λ_s initialized as $\max_{0 \leq k \leq K} \xi_{sk} - \rho$.

Since ω is very small, it is rare that condition (b) is not met, especially when ρ is chosen not to be an integer. Condition (a) is not met (all $\xi_{sk} < \rho$) when (c) $\boldsymbol{u}_s$ is an error UMI incorrectly included in $\mathcal{U}$ or (d) $\boldsymbol{u}_s$ is a valid UMI that was only weakly amplified, probably with early-cycle PCR error(s) to haplotype(s) $\boldsymbol{h}_k$ identical or similar to haplotypes included in $\mathcal{H}$. The first problem (c) may be

resolved by applying a penalty to η_s, though we did not attempt such a solution in DAUMI since we took other precautions to limit the inclusion of false UMIs in $\mathcal{U}$. The second problem (d) is difficult to resolve without properly modeling the PCR error and amplification process. DAUMI and all compared methods do not model PCR and cannot distinguish early-cycle PCR errors from legitimate variation in the sample. Instead, these methods rely on the fact that early-cycle PCR errors are exceptionally rare compared to late-cycle PCR errors, because there is far more opportunity for error after the template has been geometrically amplified. Thus, these quantification methods detect and discard most PCR and sequencing errors, but inevitably leave a few early cycle PCR errors. In the few cases where (a) is not met, we set $\hat{\gamma}_{sk} = \mathbb{1}_{\{k=\hat{k}\}}$ for $\hat{k} = \arg\max_k \xi_{sk}$. As long as the overall M step improves equation (S1) at each iteration, the EM algorithm is still guaranteed to converge (McLachlan and Krishnan, 2008). We check for this condition and never encountered a problem in any of the real or simulation datasets analyzed.

S2 Automated Selection of Penalty Parameter ρ

To automate selection of penalty parameter ρ, we propose a simple model for the observed abundance distribution $p_{\mathrm{UMI}}(x)$ of UMIs in the dataset. This distribution is a mixture of true UMIs and error UMIs generated by PCR and sequencing errors. Our goal is to identify a threshold that distinguishes true and error UMIs.

We assume molecular abundance follows a stochastic Galton-Watson branching process (Stolovitzky and Cecchi, 1996), which is often used to model the PCR amplification process (Pflug and von Haeseler, 2018). The model assumes each molecule is independently duplicated with amplification efficiency $\chi \in (0, 1)$ per PCR cycle. Let ϵ denote the PCR error rate, a small probability that a molecule is copied with at least one error. The number of *error-free* copies X_t of a true UMI without collision after the tth PCR cycle is given by the stochastic recursion

$$X_0 = 1, \quad X_t = X_{t-1} + \mathrm{Bin}(X_{t-1}, [1-\epsilon]\chi),$$

where $[1-\epsilon]\chi$ is the *error-free* amplification efficiency. After σ cycles of PCR, the molecules are sampled and sequenced to produce observed count

$$X_{\sigma+1} \sim \mathrm{Bin}(X_\sigma, [1-\delta]\iota),$$

where ι is the sampling rate and δ is the sequencing error probability.

The probability $p_{\text{BP}}(x; \sigma, \chi, \epsilon, \iota, \delta)$ of x faithful copies of a molecule after σ cycles of PCR amplification followed by sequencing is not analytically available, but can be computed by numerical methods (Lange, 2010). Assuming all PCR amplification errors are unique, the abundance of an error UMI depends only on the random PCR cycle T in which it originated and not also on the amplification of similar molecules in a neighborhood around it. Then, using the law of total probability, the probability of observing x copies of an error UMI is

$$\sum_{1 \leq t \leq \sigma} p_{\text{BP}}(x; \sigma - t, \chi, \epsilon, \iota, \delta) \Pr(T = t) + \mathbb{1}_{\{x=1\}} \Pr(T = \sigma + 1),$$

where $\Pr(T = t)$ is the probability of a PCR error in the tth cycle or a sequencing error when $T = \sigma + 1$.

Overall, the abundance of a UMI follows a mixture distribution

$$p_{\text{UMI}}(x) = p_{\text{BP}}(x; \sigma, \chi, \epsilon, \iota, \delta) \Pr(T = 0) + \sum_{1 \leq t \leq \sigma} p_{\text{BP}}(x; \sigma - t, \chi, \epsilon, \iota, \delta) \Pr(T = t) + \mathbb{1}_{\{x=1\}} \Pr(T = \sigma + 1),$$

where we have included error-free amplification and sequencing of original molecules present at $T = 0$. In practice, we take set $\iota = 1$ when calculating $p_{\text{UMI}}(x)$. In our experience, ι is substantially confounded with χ and σ, when the latter is unknown. Setting $\iota = 1$ constrains the parameters sufficiently to produce good estimates, as assessed on simulation data. Assuming UMIs are unique prior to amplification (no collision), we have

$$\Pr(T = t) \propto \begin{cases} 1 & t = 0 \\ \epsilon(1 + \chi)^{t-1} & 1 \leq t \leq \sigma \\ \delta(1 + \chi)^{\sigma} & t = \sigma + 1, \end{cases}$$

so $p_{\text{UMI}}(x)$ is a function of the parameters $\{\chi, \sigma, \epsilon, \delta\}$, which we can optimize to match the observed UMI abundance distribution.

There is no analytical solution to estimate the parameters $\{\chi, \sigma, \epsilon, \delta\}$, and the presence of unmodeled artefacts, like collision, in the right tail of the abundance distribution makes numerical maximum likelihood estimation difficult. Instead, we right-truncate the data at count τ and select parameters that minimize the Kolmogorov-Smirnov (KS) statistic (Stephens, 1974) for conditional distribution

$$p_{\text{UMI}}(x \mid x \leq \tau) = \Pr(X_{\omega+1} = x \mid X_{\omega+1} \leq \tau).$$

From the left, the first mode of the observed UMI abundance distribution represents errors, while the

second and possibly additional modes represent PCR amplified products (Stolovitzky and Cecchi, 1996; Pflug and von Haeseler, 2018). To estimate the parameters of the PCR process, it is important to retain information about PCR amplification by truncating the distribution to the right of the second mode. We use R package `multimode` (Ameijeiras-Alonso *et al.*, 2021) to find the mode of each distribution after removing sequencing errors with AmpliCI. These distributions are shown for simulation data in Figure S4 and HIV data in Figure S6. Given the conditional cumulative distribution function $F(x) = \sum_{y=0}^{x} p_{\text{UMI}}(y \mid y \leq \tau)$ and the empirical conditional distribution function $F_{n(\tau)}(x) = \frac{1}{n(\tau)} \sum_{s=1}^{n(\tau)} \mathbb{1}_{\{X_s \in [0,x]\}}$ for $n(\tau)$ observed UMIs $\boldsymbol{b}_s$ with abundance $X_s = \sum_{i=1}^{n} \mathbb{1}_{\{\boldsymbol{b}_i = \boldsymbol{b}_s\}} \leq \tau$, the KS statistic is the largest absolute difference between these two distributions, $\sup_{x \in \{0,1,2,\dots\}} |F_{n(\tau)}(x) - F(x)|$. Specifically, we use a grid search over $\tau \in \{100, 150, 200, 300\}$, $\chi \in \{0.05, 0.10, \dots, 1\}$, $\sigma \in \{6, 7, \dots, 21\}$, $\epsilon \in \{0.0005, 0.0010, \dots, 0.005\}$ and $\delta \in \{0.005, 0.006, \dots, 0.014\}$. The ranges are selected based on the values observed in real experiments (Schirmer *et al.*, 2016; Stolovitzky and Cecchi, 1996; Potapov and Ong, 2017; Quince *et al.*, 2011), but the user may need to adjust these parameters, particularly τ or σ, to match their data. Note, σ is not necessarily the actual number of experimental PCR cycles, but an *effective* number of PCR amplification cycles under our "ideal" model of PCR amplification. Rather than an exhaustive search over the entire grid, we implement a block relaxation algorithm (de Leeuw, 1994), alternating between optimization of (χ, σ) while holding (ϵ, δ) fixed and vice versa until the KS statistic converges with the relative change between iterations $< 10^{-4}$ for each possible value of τ.

The remaining challenge is to choose a threshold ρ that will remove most error UMIs without discarding true UMIs. In practice, we choose ρ to be the 5th percentile of the fitted observed abundance distribution $p_{\text{BP}}(x; \hat{\sigma}, \hat{\chi}, \hat{\epsilon}, 1, \hat{\delta})$ for true UMIs, which implies an estimated five percent of true variants will have observed abundance below the threshold ρ. Table S4 shows the fitted model and selected ρ for each $\tau \in \{100, 150, 200, 300\}$ in all test datasets. For most datasets, our strategy works well and helps to select appropriate ρ (Figure S3 and S5). On the V1V2 and V3 datasets, the strategy selects an optimal or near optimal ρ (Table S9), but that choice is always $\rho = 1$ for V3, no matter the truncation point τ. There is no obvious second mode in raw UMI count data (Figure S5), either because there was very inefficient amplification, we estimate $\hat{\chi} = 0.05$, or severe downsampling at sequencing. Both scenarios limit UMI replication and inhibit all UMI-based quantification methods. At the same time, these data display some very highly replicated UMIs, perhaps because of high rates of UMI collision or highly stochastic amplification rates (Potapov and Ong, 2017). For all these reasons, it is difficult to pick a threshold to separate errors from real variants. In general, we suggest visual assessment of the observed UMI abundances before and after error removal to assess the adequacy of the automatically selected ρ. In the case of V3, we manually chose $\rho = 20$, which yielded slightly worse performance in our assessments than the automatically selected $\rho = 1$ (Table S9), thus demonstrating that we did not

cherry pick results. The good news for the user is that DAUMI performance is minimally impacted by selection of ρ. It appears to have most impact on low amplification datasets like V3, but even with our suboptimal manual override choice of ρ, DAUMI performance was superior to other methods (Table 1).

For assessing performance on the real data, we had to choose ρ for random binary splits of the data (§3.2). For V1V2, ρ was selected independently for each subset by the automatic procedure. For V3, $\rho = 10$ was selected for both subsets, half the value chosen by visual assessment on the whole dataset. For the single-cell molecular spike dataset, we pooled the UMIs from all cells and estimated $\rho = 9$ by the automatic procedure. Since we expect minimal UMI collision across cells, we applied this threshold directly to each cell.

S3 Tuning Run Parameters of Other Methods

As just demonstrated for selection of ρ, it is important and sometimes difficult to set appropriate values for run parameters; the default values are not always optimal. To verify the optimality of the default parameters used in the *other* quantification methods studied in this work, we ran Calib, UMI-tools and DAUMI under different parameter settings. There are no run parameters for the Naïve method, and tuning Starcode-umi was deemed too difficult as there are at least five parameters and no guidance to set them.

Table S6 shows the results on simulation data while varying run parameters. Specifically, we tried edit distance $d = 1$ or $d = 2$ for UMI-tools, varied ρ for DAUMI, and tried six parameter settings for Calib as suggested by the authors (Orabi *et al.*, 2018). UMI-tools performed better with the default edit distance $d = 1$, though the number of false positives slightly increases. While Calib's default parameter values did not always produce the best results, the effect of different parameter settings was minimal. Finally, we show that DAUMI at default settings always outperformed other algorithms at their optimal parameter settings, even though default ρ does not always achieve the best performance.

S4 Abundance Estimation for Known Variants

In some cases, the set of possible haplotypes is known a priori (Zhang *et al.*, 2021). To mimic this situation, we set $\mathcal{H}$ to the true 25 haplotypes for simulations 1–4. This experiment also allows us to explore DAUMI abundance estimation with less chance for false positives, since only a few of the 25 true haplotypes are not sampled in the data and could be incorrectly included as false positives. Table S7 shows that performance does increase as compared to the performance in Table S6, where the set $\mathcal{H}$ is not known. DAUMI can accurately estimate deduplicated abundance on datasets *without* UMI collision

(Simulations 1–2) as expected, and there is little difference as ρ varies. However, for datasets *with* UMI collision (Simulations 3–4), DAUMI underestimates haplotype deduplicated abundance (regression slope $b < 1$), especially as ρ increases, because then even legitimate UMI to haplotype linkages with transition probability $\gamma_{sk} > 0$ are eliminated by the penalty. For Simulation 4 with low PCR efficiency, the underestimation is particularly sensitive to ρ, presumably because there are more true molecules failing to amplify well. When two haplotypes are assigned to the same UMI and the number of assigned reads to one haplotype is below the threshold ρ, DAUMI will eliminate the less observed haplotype as a likely error, leading to an underestimation of haplotype abundance. However, it is not a good idea to simply set ρ very low, since then DAUMI will retain false linkages between UMIs and haplotypes. There may be weak evidence for UMI/haplotype combinations $(\boldsymbol{u}_s, \boldsymbol{h}_k)$ that do not actually exist in the sample if there is at least one observed read $(\boldsymbol{b}_i, \boldsymbol{r}_i)$ plausibly generated from $(\boldsymbol{u}_s, \boldsymbol{h}_k)$. For example, DAUMI identifies two false positives for Simulation 3 and one false positive for Simulation 4 with $\rho = 0.01$ (a choice well below the automatically selected value [see Table S2]). These are haplotypes included in $\mathcal{H}$ that were not actually sampled. When the haplotype set $\mathcal{H}$ is not constrained by a known truth, there will be even more weak linkages that survive when ρ is set too low.

S5 Extracting UMIs from V1V2 Dataset

The reverse reads of the V1V2 dataset contain a 52–55 nucleotide (nt) technical sequence at the 5' end, starting with 0–3 random nucleotides, an 18nt adapter, a 9nt UMI, and a 25nt primer with two ambiguous nucleotides, one Y and another R, used to amplify the target region in the HIV *env* gene. Our goal is to extract the 9nt UMI, discard the rest of the technical sequence, and recover the approximately 250nt sampled sequence. This section describes an independent model used only in data preprocessing; reuse of DAUMI model notation does not communicate simile.

We assume there are no indel errors in the technical sequence of each read, so the adapter sequence starts at read position 1, 2, 3, 4, or is not present at all. Let $W_{i1} \in \{0, 1, 2, 3, 4\}$ be the unknown number of random nucleotides at the start of read $\boldsymbol{X}_i$ or $W_{i1} = 4$ when the technical sequence is not present. Further, let $W_{i2} \in \{0, 1, 2, 3\}$, defined only when $W_{i1} < 4$, be the unknown state of the *unambiguous* primer sans UMI with the Y and R nucleotides resolved. We assume W_{i1} and W_{i2} are independent and define $\eta_k = \Pr(W_{i1} = k)$ and $\zeta_l = \Pr(W_{i2} = l \mid W_{i1} < 4)$. The bivariate $\boldsymbol{W}_i = (W_{i1}, W_{i2})$ is unobserved.

For the observed data, dropping read index i, let

$$p_{jx}(\boldsymbol{w}) = \Pr(X_j = x \mid \boldsymbol{W} = \boldsymbol{w})$$

be the probability of read nucleotide x at position j given $\boldsymbol{W} = \boldsymbol{w}$. Read position j can index the 0–3 nucleotides in the 5' random leader, nucleotides in the UMI, either of two ambiguous positions in the primer, other unambiguous nucleotides in the technical sequence or the sample sequence. Assume the random 5' leader, the UMI and the sampled sequence are adequately modeled as independently and identically distributed nucleotides within class, but allow the nucleotide composition to vary: $\boldsymbol{q}_r = (q_{r\mathrm{A}}, q_{r\mathrm{C}}, q_{r\mathrm{G}}, q_{r\mathrm{T}})$ for the random nucleotides in the 5' leader and UMI and $\boldsymbol{q}_s = (q_{s\mathrm{A}}, q_{s\mathrm{C}}, q_{s\mathrm{G}}, q_{s\mathrm{T}})$ for the sample sequence. At all other sites, assume errors are independent, but not equally likely across sites. Let δ_j be the probability of an error-free nucleotide at read position j. Given an error, let $\gamma_{\mathrm{N}_1\mathrm{N}_2}$ be the probability that true nucleotide N_1 is misread as read nucleotide N_2. Define the index sets $\mathcal{V} = \{19, 20, \ldots, 27\}$ for the UMI indices, $\mathcal{T} = \{1, 2, \ldots, 52\} \backslash \{\mathcal{V}, 35, 42\}$ for the unambiguous technical sequence, and $\mathcal{S} = \{53, 54, \ldots\}$ for the sample sequence. Let $\boldsymbol{D} = (D_1, D_2, \ldots, D_{52})$ be the 52 nucleotides in the technical sequence, excluding the 5' leader, Nucleotides $D_{35} = \mathrm{R}$ and $D_{42} = \mathrm{Y}$ are ambiguous, so let $R_l, Y_l \in \{\mathrm{A}, \mathrm{C}, \mathrm{G}, \mathrm{T}\}$ be the resolved nucleotides when $W_2 = l$, and $\mathbb{1}_C(x) = \mathbb{1}_{\{x \in C\}}$ indicate the event $x \in C$. Still dropping read index i, we have

$$
\begin{aligned}
p_{jx}(\boldsymbol{w}) &= \Pr(X_j = x | \boldsymbol{W} = \boldsymbol{w}) \\
&= (q_{sx})^{\mathbb{1}_{\{w_1=4\}}} \Bigg[(q_{bx})^{\mathbb{1}_{\{j \le w_1\}} + \mathbb{1}_{\mathcal{V}}(j-w_1)} (q_{sx})^{\mathbb{1}_{\mathcal{S}}(j-w_1)} \\
&\quad \left(\delta_j^{\mathbb{1}_{\{x=Y_l\}}} [(1-\delta_j)\gamma_{Y_l x}]^{\mathbb{1}_{\{x \neq Y_l\}}} \right)^{\mathbb{1}_{\{35\}}(j-w_1)} \left(\delta_j^{\mathbb{1}_{\{x=R_l\}}} [(1-\delta_j)\gamma_{R_l x}]^{\mathbb{1}_{\{x \neq R_l\}}} \right)^{\mathbb{1}_{\{42\}}(j-w_1)} \\
&\quad \left(\delta_j^{\mathbb{1}\left\{x = D_{(j-w_1)}\right\}} [(1-\delta_j)\gamma_{D_{(j-w_1)}x}]^{\mathbb{1}\left\{x \neq D_{(j-w_1)}\right\}} \right)^{\mathbb{1}_{\mathcal{T}}(j-w_1)} \Bigg]^{\mathbb{1}_{\{w_1 \le 3\}}} .
\end{aligned}
$$

Let $\boldsymbol{\delta} = (\delta_1, \delta_2, ...)^T$, $\boldsymbol{\gamma} = (\gamma_{\mathrm{AC}}, \gamma_{\mathrm{AG}}, \gamma_{\mathrm{AT}}, \gamma_{\mathrm{CA}}, \gamma_{\mathrm{CG}}, \gamma_{\mathrm{CT}}, \gamma_{\mathrm{GA}}, \gamma_{\mathrm{GC}}, \gamma_{\mathrm{GT}}, \gamma_{\mathrm{TA}}, \gamma_{\mathrm{TC}}, \gamma_{\mathrm{TG}})$, $\boldsymbol{\eta} = (\eta_0, \eta_1, \ldots, \eta_4)$, and $\boldsymbol{\zeta} = (\zeta_0, \zeta_1, \zeta_2, \zeta_e)$. Then our unknown parameter vector is $\boldsymbol{\theta} = (\boldsymbol{\delta}^T, \boldsymbol{\gamma}^T, \boldsymbol{q}_u^T, \boldsymbol{q}_s^T, \boldsymbol{\eta}^T, \boldsymbol{\zeta}^T)^T$. Finally, if there are n reads, the length of read i is J_i, all reads are independent and all nucleotides within reads are conditionally independent, then the complete data likelihood is

$$
L_C(\boldsymbol{\theta} \mid \boldsymbol{X}, \boldsymbol{W}) = \prod_{i=1}^{n} \Pr(\boldsymbol{X}_i = \boldsymbol{x}_i, \boldsymbol{W}_i = \boldsymbol{w}_i) = \prod_{i=1}^{n} \prod_{k=0}^{4} \prod_{l=0}^{3} \Big[\Pr(W_{i1} = k, W_{i2} = l) \prod_{j=1}^{J_i} p_{X_{ij}}(k, l) \Big]^{\mathbb{1}_{\{W_{i1}=k, W_{i2}=l\}}} .
$$

In the E step, we need to compute $\mathbb{E}[\ln L_C(\boldsymbol{\theta} \mid \boldsymbol{X}, \boldsymbol{W}) \mid \boldsymbol{X}]$, but since the complete data log likelihood

is linear and the reads are independent, it amounts to computing

$$\begin{aligned} e_{ikl} &:= \Pr\left(W_{i1} = k, W_{i2} = l \mid \boldsymbol{X}_i\right) \\ &\propto \eta_k \zeta_l^{\mathbb{1}_{\{k<4\}}} \prod_{j=1}^{J_i} p_{jX_{ij}}(l, k), \end{aligned}$$

for each $i \in \{1, 2, \ldots, n\}$, $k \in \{0, 1, 2, 3\}$, and $l \in \{0, 1, 2, 3\}$. When $k = 4$, l is undefined, and we have $e_{i4} = \Pr(W_{i1} = 4 \mid \boldsymbol{X}_i)$. The update equations in the M step are

$$\begin{aligned} \eta_k &= \frac{n_{\eta k}}{\sum_l n_{\eta l}} & \zeta_l &= \frac{n_{\zeta l}}{\sum_{k=0}^{3} n_{\zeta k}} \\ q_{ux} &= \frac{n_{ux}}{\sum_y n_{uy}} & q_{sx} &= \frac{n_{sx}}{\sum_y n_{sy}} \\ \delta_j &= \frac{n_{j0}}{n_{j0} + n_{j1}} & \gamma_{yx} &= \frac{n_{yx}}{\sum_w n_{yw}}, \end{aligned}$$

with expected counts

$$\begin{aligned} n_{\eta k} &= \begin{cases} \sum_{i=1}^{n} e_{ikl} & k < 4 \\ \sum_{i=1}^{n} e_{ik} & k = 4 \end{cases} \\ n_{\zeta l} &= \sum_{i=1}^{n} e_{ikl}, k < 4 \\ n_{ux} &= \sum_{i=1}^{n} \sum_{k=0}^{3} \sum_{l=0}^{3} e_{ikl} \sum_{j=1}^{J_i} \mathbb{1}_{\{X_{ij}=x\}} (\mathbb{1}_{\{j \le k\}} + \mathbb{1}_{\mathcal{V}}(j-k)) \\ n_{sx} &= \sum_{i=1}^{n} [\sum_{k=0}^{3} \sum_{l=0}^{3} e_{ikl} \sum_{j=k+52}^{J_i} \mathbb{1}_{\{X_{ij}=x\}} + e_{i4} \sum_{j=1}^{J_i} \mathbb{1}_{\{X_{ij}=x\}}] \\ n_{j0} &= \sum_{i=1}^{n} \sum_{k=0}^{3} \sum_{l=0}^{3} e_{ikl} \mathbb{1}_{\{X_{i,j+k}=D_j\}} \left[\mathbb{1}_{\mathcal{T}}(j-k) + \mathbb{1}_{\{35\}}(j-k) + \mathbb{1}_{\{42\}}(j-k)\right] \\ n_{j1} &= \sum_{i=1}^{n} \sum_{k=0}^{3} \sum_{l=0}^{3} e_{ikl} \mathbb{1}_{\{X_{i,j+k} \neq D_j\}} \left[\mathbb{1}_{\mathcal{T}}(j-k) + \mathbb{1}_{\{35\}}(j-k) + \mathbb{1}_{\{42\}}(j-k)\right] \\ n_{yx} &= \sum_{i=1}^{n} \sum_{k=0}^{3} \sum_{l=0}^{3} e_{ikl} \sum_{j=1}^{J_i} \mathbb{1}_{\{X_{i,j+k}=x, D_j=y\}} \left[\mathbb{1}_{\mathcal{T}}(j-k) + \mathbb{1}_{\{35\}}(j-k) + \mathbb{1}_{\{42\}}(j-k)\right]. \end{aligned}$$

We fit all 61,881 reads to this model, converting ambiguous N nucleotides (affecting just 170 reads) to A. We iterated the EM algorithm until the relative change in log likelihood was below 0.001. We dropped 18,861 reads shorter than the 52nt primer and 700 reads unlikely to contain the technical sequence, *i.e.* with $\Pr(W_{i1} = 4 \mid \boldsymbol{X}_i) \geq 0.5$. We dropped three reads with posterior probability $\Pr(W_{i1} = 4 \mid \boldsymbol{X}_i) < 0.5$, but $\arg\max_{k \in \{0,1,2,3\}, l \in \{0,1,2,3\}} \Pr(W_{i1} = k, W_{i2} = l \mid \boldsymbol{X}_i) < \Pr(W_{i1} = 4 \mid \boldsymbol{X}_i)$; these could have been

retained but were lost to a small bug in our code. Since we noticed several reads had a poor match to the 25nt primer, we dropped another 8,763 reads where the log likelihood of the primer region only was smaller than -100 as likely technical artefacts. Finally, we dropped 25 surviving reads with ambiguous N nucleotides. For the 33,529 remaining reads, we assumed the 9nts starting at position $\hat{w}_{i1} + 18$ of the ith read constituted the UMI and the sampled molecule extended from $\hat{w}_{i1} + 52$ to the end of the read. Here, $\hat{w}_{i1} = \arg\max_{k \in \{0,1,2,3\}} \Pr(W_{i1} = k \mid \boldsymbol{X}_i)$ is the most likely number of 5' leader nucleotides. The posterior probabilities used for screening were obtained from the $e_{ikl}, k < 4$ and e_{i4} values computed in the last E step.

S6 Supplementary Figures and Tables

Here we include all supplementary figures and tables that have been cited in the main text or in supplementary sections S1–S4. Figure S1 compares DAUMI and other methods with respect to their ability to detect UMI collisions. Figure S2 shows the pipeline for simulating datasets. Figures S3–S6 illustrate the observed UMI abundance distributions before and after error correction; the selected parameter ρ is shown as a red vertical line on the UMI abundance distributions without error correction (Figures S3 and S5). Figure S7 shows the performance of UMI-unaware AmpliCI on the simulated datasets. Figure S8 compares true and estimated values of UMI mixing proportions η in simulation. Figure S9 shows the number of estimated haplotypes, their deduplicated abundances, and a Venn diagram of overlaps for the HIV V1V2 data. Figure S10 demonstrates the low UMI diversity found in the HIV V3 dataset. Figure S11 uses Venn diagrams to compare haplotypes found by different methods on the HIV V3 dataset.

Table S1 provides a comparison of methods, specifically listing what kinds of errors each method is stated to handle. Table S2 summarize the four simulation conditions that generated the data analyzed in Figure 3. Table S3 provides running time information for DAUMI. Table S4 provides detailed information about the achieved fits of the branching process model (§S2) during selection of ρ. Table S5 provides numeric summaries for the linear model fits shown in Figure 3. Table S6 shows the performance of different methods while varying run parameters on the simulation data. Table S7 shows the performance of DAUMI with $\mathcal{H}$ fixed to a set of known candidates and varying ρ on the simulation data. Table S8 summarizes the HIV datasets. Table S9 shows the effect of ρ on DAUMI performance on the HIV datasets. Table S10 compares methods when all singletons are a priori disqualified to be haplotypes, which is the default behavior for DAUMI and Naïve, but not the other methods. Table S11 shows the improvements of DAUMI with a different initial set $\mathcal{U}$ on one single cell of the single-cell molecular spikes dataset.

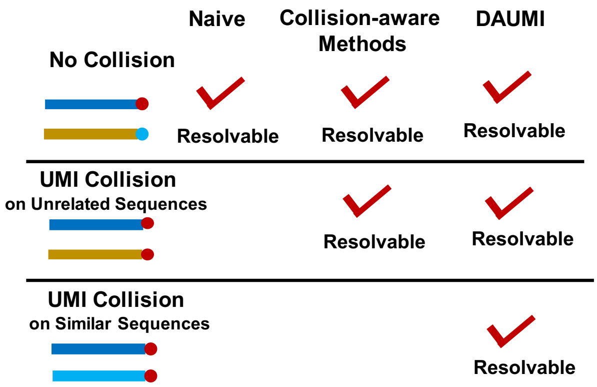

Figure S1: DAUMI can correctly resolve the origin of haplotypes even if there are UMI collisions.

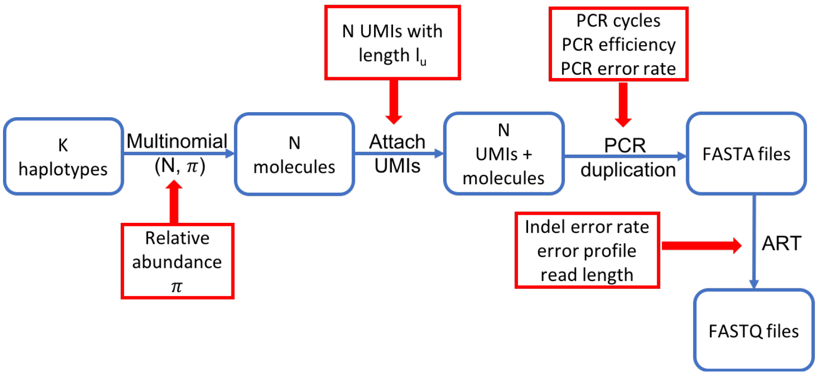

Figure S2: Data simulation pipeline. Total N molecules are tagged with UMIs, after being sampled from K haplotypes under a multinomial distribution Mult(N, π). Then tagged molecular are amplified with a given efficiency for a given number of PCR cycles to mimic PCR amplification process. Lastly, ART is applied to simulate single-end reads to generate final FASTQ files.

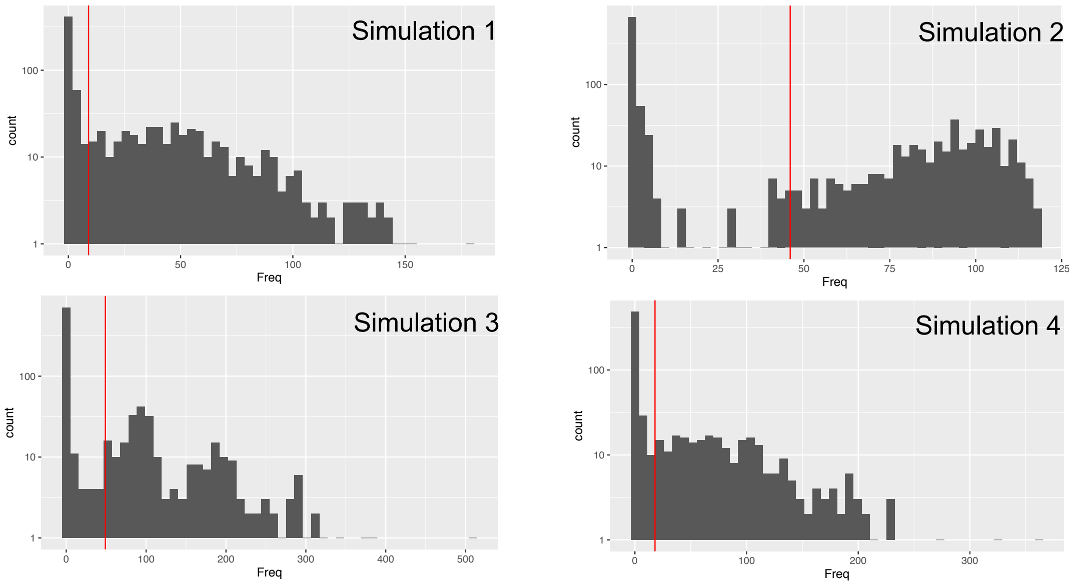

Figure S3: UMI observed abundance distribution of simulation datasets (errors uncorrected). Simulations 1 and 4 were simulated with PCR efficiency 0.5 in 10 cycles. Simulations 2 and 3 were simulated with PCR efficiency 0.9 in 7 cycles. There are UMI collisions in Simulations 3 and 4. The red vertical line is the ρ selected by our proposed method.

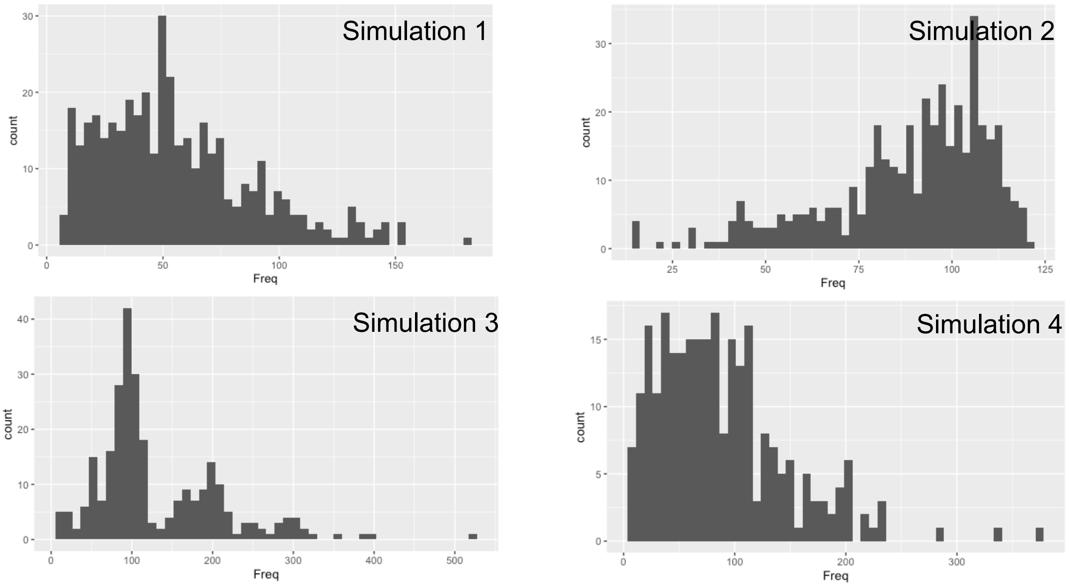

Figure S4: UMI abundance distribution of simulation datasets after removing sequencing errors with UMI-unaware AmpliCI. See legend of Figure S3 for more details about the simulations.

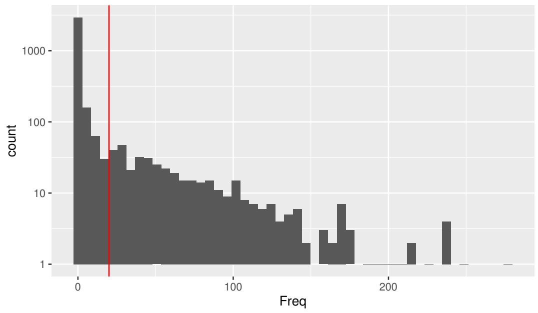

(a) VIV2

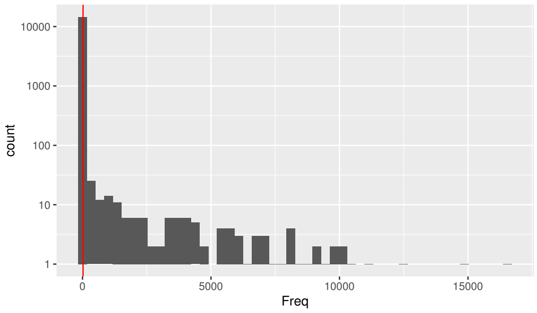

(b) V3

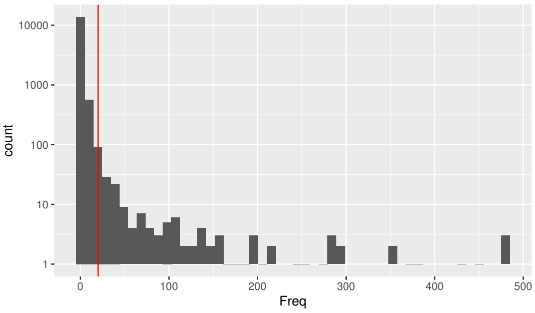

(c) V3 (Freq $\leq$ 500)

Figure S5: UMI observed abundance distribution of V1V2 and V3 datasets (errors uncorrected). V3 is highly right-skewed, so (c) focuses on the left tail for UMI with observed abundance at or below 500. The red vertical line is the ρ selected either by our proposed method (V1V2) or by eye (V3).

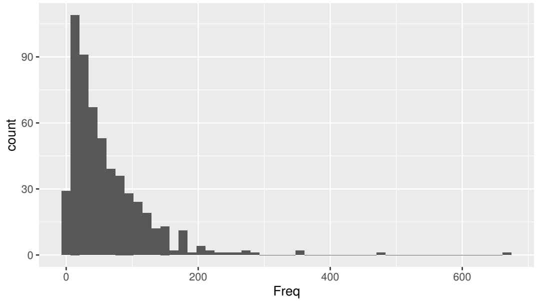

(a) VIV2

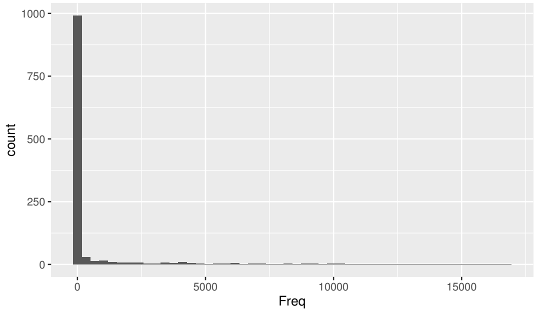

(b) V3

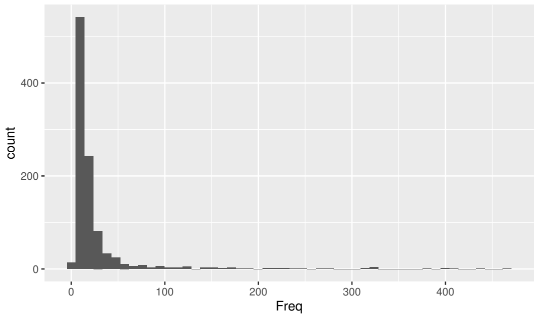

(c) V3 (Freq $\leq$ 500)

Figure S6: UMI abundance distribution of V1V2 and V3 after removing sequencing errors with UMI-unaware AmpliCI. Again, (c) focuses on the left tail of the V3 distribution.

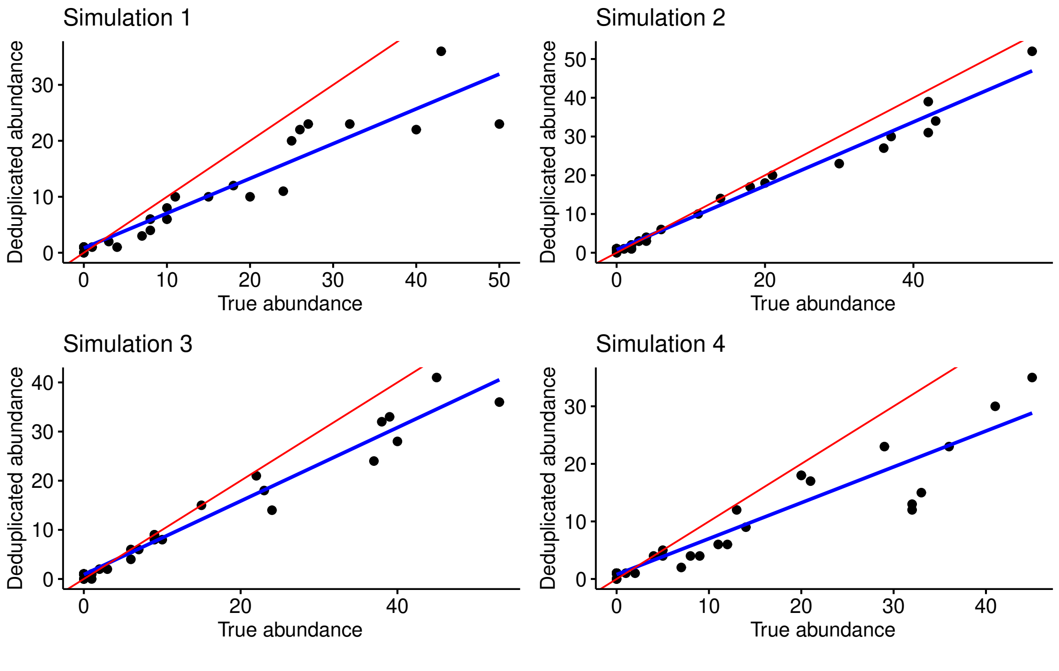

Figure S7: Abundance estimation of UMI-unaware AmpliCI on simulated data. AmpliCI was performed to denoise whole (UMI + haplotype) sequences. Deduplicated abundances were count as number of denoised haplotypes (with UMI unattached). See legend of Figure 3 for more details.

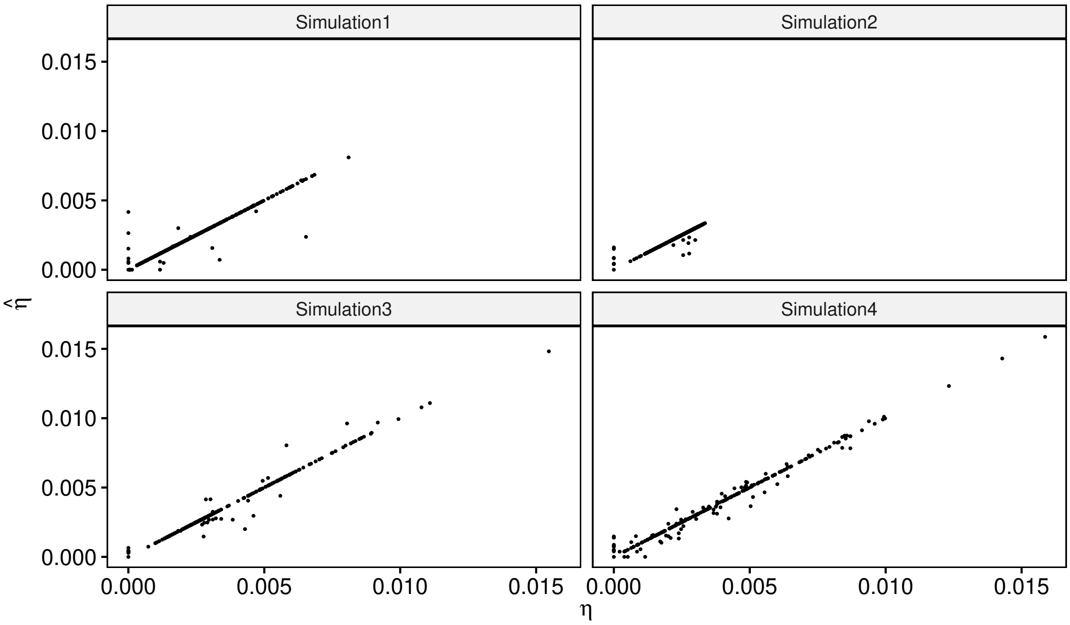

Figure S8: Scatter plots of estimated UMI mixing proportions $\hat{\boldsymbol{\eta}}$ vs. true $\boldsymbol{\eta}$ on four simulation datasets. Accuracy of transition matrix $\hat{\boldsymbol{\Gamma}}$ is demonstrated in Figure 3.

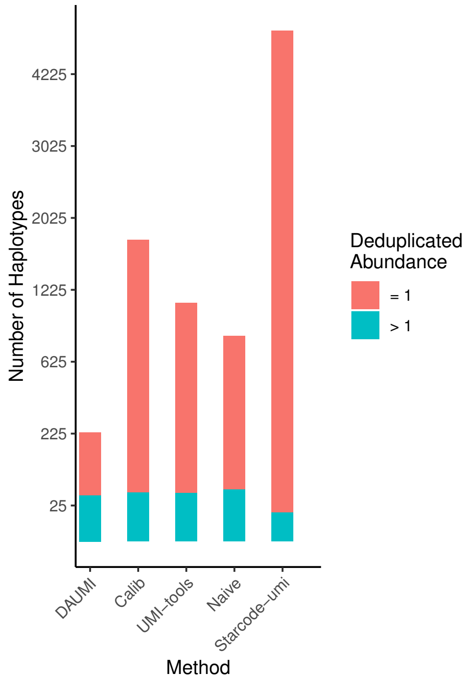

(a)

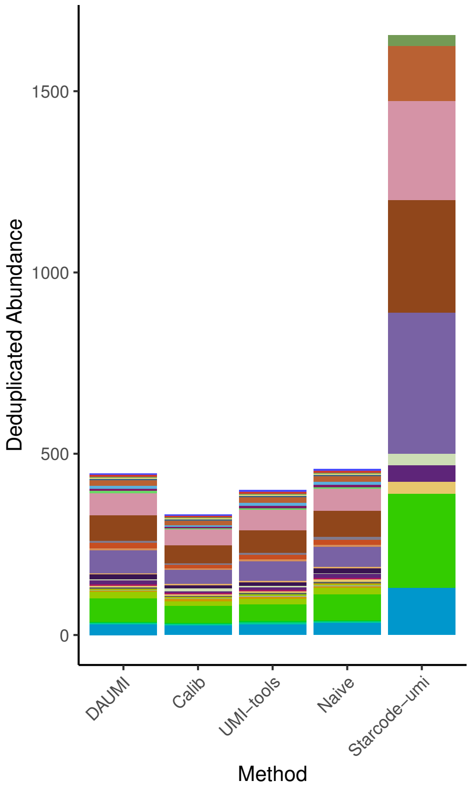

(b)

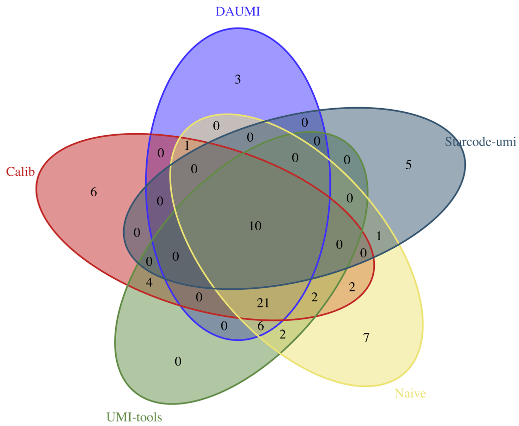

(c)

Figure S9: Results for V1V2 data (Bhiman *et al.*, 2015) by DAUMI ($\rho = 20$), Calib, UMI-tools, Starcode-umi and Naïve methods. (a) Total number of recovered haplotypes, plotted on square root scale. (b) Estimated deduplicated abundance of the 31 haplotypes identified by all methods except Starcode-umi. (c) Venn Diagram of recovered haplotypes with deduplicated abundance ≥ 2, made by VennDiagram R package (v1.6.20) (Chen and Boutros, 2011).

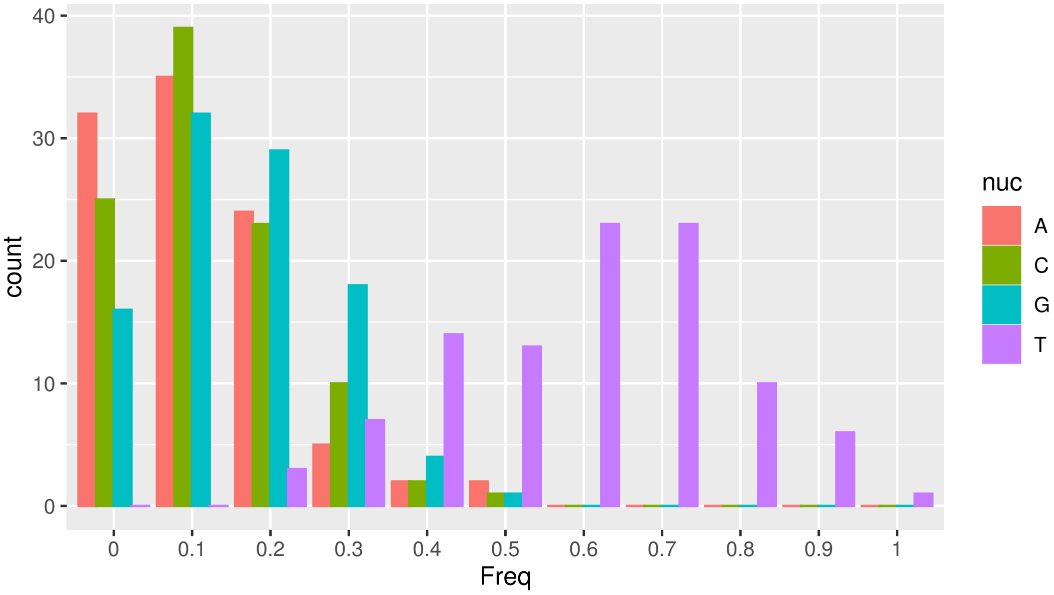

Figure S10: Nucleotide usage frequency per UMI in abundant UMIs of V3 dataset. The UMIs are of length 10nt in this dataset. We only take into account the top 100 UMIs, thus the total counts for each color should be equal to 100.

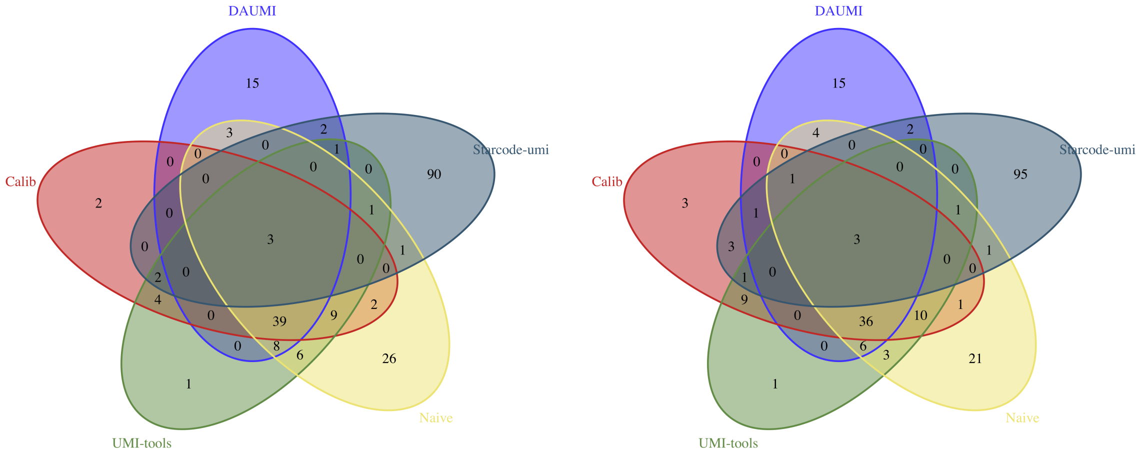

(a) Subset 1

(b) Subset 2

Figure S11: Venn diagrams comparing recovered haplotypes (with deduplicated abundance no less than two) on two subsets of V3 (Caskey *et al.*, 2017) by DAUMI ($\rho = 10$), Calib, UMI-tools, Starcode-umi and Naïve methods, made by VennDiagram R package (v1.6.20) (Chen and Boutros, 2011). Results of the first out of five random partitions is shown.

Table S1: Which sofware can remove substitution, indertion/deletion (Indel), recombination, and collision errors during PCR or sequencing (Seq) in UMI or sample sequence (Sample). Ability confers no guarantee; details in discussion.

	**Introduced**		**Handled by**[1]					
**Error**	**in**	**during**	**DA**	**AC**	**UT**	**Naïve**	**Calib**	**Star**
Substitution	UMI	PCR, Seq	Seq[2]	NA[3]	Yes	No	Yes	Yes
Substitution	Sample	PCR, Seq	Yes	Yes	UMI[4]	UMI[4]	Yes	Yes
Indel	UMI	PCR, Seq	Init[5]	NA	Yes	No	Yes	Yes
Indel	Sample	PCR, Seq	Yes	Yes	UMI	UMI	Yes	Yes
All	Sample	pre-PCR[6]	No	No	No	No	No	No
Recombination	All	PCR	No	No	UMI	UMI	No	No
Collision	NA	NA	Yes	NA	No	No	Yes	Yes

[1]DA: DAUMI; AC: AmpliCI; UT: UMI-tools; Star: Starcode-umi. [2]Initialization removes rare PCR errors in UMI. [3]Not Applicable. [4]UT, Naïve may correct errors in sample attached to the clustered or same UMI. [5]Initialization method can remove UMI indel errors. [6]Errors before PCR, *i.e.* during cDNA synthesis, are difficult to distinguish from rare variants.

Table S2: Simulated datasets. σ: number of PCR cycles; χ: PCR efficiency; Mean: expected amplified abundance $(1+\chi)^\sigma$; Var.: variance in amplified abundance $(1-\chi)(1+\chi)^\sigma((1+\chi)^\sigma-1)/(1+\chi)$ (Pflug and von Haeseler, 2018); Hap.: number (of 25) true haplotypes sampled, with number of unsampled haplotypes (true abundance 0) in parenthesis; UMI: number true unique UMIs; Mol.: number unique sampled molecules; ρ: selected penalty parameter.

**Simulation**	σ	χ	**Mean**	**Var.**	**Hap.**	**UMI**	**Mol.**	ρ
1	10	0.5	57.67	1089.20	22 (3)	400	400	9
2	7	0.9	89.39	415.83	23 (2)	400	400	45
3	7	0.9	89.39	415.83	22 (3)	263	400	49
4	10	0.5	57.67	1089.20	22 (3)	257	400	18

Table S3: DAUMI runtimes on tested datasets. Time was recorded by GNU Time on a Mac Air for the whole pipeline, that include initialization. No. Reads: total number of reads; No. Mol.: total number of molecules before amplification; No. Hap.: number of haplotypes in $\mathcal{H}$; No. Iter.: number of EM iterations. For the single-cell molecular spikes dataset, DAUMI was run on each cell independently. All cells (20k–300k reads/cell) were run in parallel on a distributed server and compute times varied. For comparable timing, we selected one cell with 25k reads to run on the Mac Air.

**Dataset**	**No. reads**	**No. Mol.**	**No. Hap.**	**No. Iter.**	**Time**
Simulations	22k, 35k	400	∼25	∼5	<2min
HIV V1V2 subsets	17k	∼500	∼100	∼10	<2min
HIV V3 subsets	265k	∼800	∼200	∼20	2hr
One molecular spikes dataset	25k	∼1800	∼1800	5	1hr 30min

Table S4: Selection of right-tail truncation point τ when modeling UMI abundance distribution. To remove the unmodeled UMI collisions, we only retain UMI with observed counts $X \leq \tau$. Trunc.: truncation point τ; Eff.: estimated PCR amplification efficiency $\hat{\chi}$; Cycles: estimated effective number $\hat{\sigma}$ of PCR cycles; PCR Err.: estimated probability $\hat{\epsilon}$ of PCR error; Seq. Err.: estimated probability $\hat{\delta}$ of sequencing error. Thres.: selected penalty parameter ρ. KS: Kolmogorov-Smirnoff goodness-of-fit test statistic, minimized to select the truncation point. Mode is the estimated mode of the observed abundance distribution after removing error UMIs with AmpliCI. To eliminate the impact of long right tail, extreme outliers, values above $Q3 + 3 \times IQR$ were removed for mode detection. The row of the selected truncation point and minimum KS is bolded for each dataset.

**Datasets**	**Trunc.** τ	**Eff.** $\hat{\chi}$	**Cycles** $\hat{\sigma}$	**PCR Err.** $\hat{\epsilon}$	**Seq. Err.** $\hat{\delta}$	**Thres.** ρ	**KS**
Simulation 1	**100**	**0.45**	**11**	**0.0035**	**0.013**	**9**	**0.0194**
(mode: 45)	150	0.45	11	0.0040	0.013	10	0.0213
	200	0.45	11	0.0040	0.013	10	0.0246
	300	0.45	11	0.0040	0.013	10	0.0249
Simulation 2	100	0.75	8	0.0050	0.014	25	0.0511
(mode: 102)	150	0.90	7	0.0050	0.014	45	0.0307
	200	0.90	7	0.0050	0.014	45	0.0307
	**300**	**0.90**	**7**	**0.0050**	**0.014**	**45**	**0.0307**
Simulation 3	**100**	**0.95**	**7**	**0.0050**	**0.012**	**49**	**0.0145**
(mode: 96)	150	0.65	10	0.0050	0.012	30	0.0443
	200	0.60	11	0.0035	0.010	34	0.0486
	300	0.50	12	0.0040	0.014	24	0.0361
Simulation 4	**100**	**0.60**	**10**	**0.0045**	**0.009**	**18**	**0.0201**
(mode: 66)	150	0.60	10	0.0050	0.012	30	0.0310
	200	0.35	15	0.0030	0.014	11	0.0215
	300	0.35	15	0.0030	0.014	74	0.0249
VIV2	**100**	**0.60**	**11**	**0.0045**	**0.010**	**20**	**0.0180**
(mode: 49)	150	0.70	10	0.0050	0.012	37	0.0300
	200	0.60	12	0.0045	0.008	39	0.0325
	300	0.70	11	0.0050	0.011	72	0.0440
V3	100	0.05	10	0.0050	0.014	1	0.0399
(mode: 12)	150	0.05	10	0.0050	0.014	1	0.0392
	200	0.05	10	0.0050	0.014	1	0.0387
	**300**	**0.05**	**10**	**0.0050**	**0.014**	**1**	**0.0382**

Table S5: Abundance estimation for simulated data. From linear model Deduplicated Abundance = $b \times$ True Abundance fit, we report b: coefficient, > 1 for overestimation, < 1 for underestimation; RSS: residual sum of squares, 0 for perfect estimation; R^2: proportion of variance explained, 1 for perfect estimation. The best achieved metric (per column) is bolded per simulation.

**Method**	$\boldsymbol{b}$	**RSS**	$\boldsymbol{R^2}$	$\boldsymbol{b}$	**RSS**	$\boldsymbol{R^2}$
	**Simulation 1**			**Simulation 2**		
Calib	0.843	79.077	0.990	0.884	71.070	0.993
DAUMI	0.974	**39.656**	**0.996**	**0.995**	**22.691**	**0.998**
Naïve	**0.997**	110.909	0.990	1.063	108.241	0.993
Starcode-umi	1.232	431.900	0.975	1.411	1315.705	0.953
UMI-tools	0.931	48.449	0.995	0.963	32.484	0.997
	**Simulation 3**			**Simulation 4**		
Calib	0.694	327.521	0.951	0.651	303.096	0.940
DAUMI	**0.932**	**29.173**	**0.997**	**0.880**	**96.708**	**0.989**
Naïve	0.665	243.311	0.960	0.639	217.475	0.954
Starcode-umi	1.236	717.798	0.964	1.223	883.979	0.947
UMI-tools	0.549	135.946	0.967	0.538	120.385	0.964

Table S6: Effects of run parameters on performance of DAUMI, UMI-tools, and Calib for simulated data. Underlined numbers indicate the default parameter settings, and the best performance for each method per dataset and metric are bolded, unless there is a tie. For parameters, d: threshold of edit distance between UMIs, e: error tolerance, k: k-mer size, m: number of minimizer, t: minimizer threshold, ρ: penalty parameter. Six parameter sets were recommended by Calib for datasets with mean read length 250 and mean barcode length 4 (Orabi *et al.*, 2018). TP: number of true haplotypes recovered. FP: false positives. Other columns are described in the Table S5.

Datasets	Method	Parameters	FP	TP	b	RSS	R^2
Simulation 1	UMI-tools	$\underline{d = 1}$	27	22	**0.931**	**48.450**	**0.995**
	UMI-tools	$d = 2$	**23**	22	0.788	67.244	0.990
	Calib	$e = 1; k = 8; m = 5; t = 2$	29	22	0.840	74.409	0.991
	Calib	$e = 1; k = 8; m = 6; t = 2$	**28**	22	0.846	73.409	0.991
	Calib	$e = 1; k = 8; m = 6; t = 3$	**28**	22	0.846	**66.775**	**0.992**
	Calib	$\underline{e = 1; k = 8; m = 7; t = 2}$	**28**	22	0.843	79.077	0.990
	Calib	$e = 1; k = 8; m = 7; t = 3$	**28**	22	0.843	79.077	0.990
	Calib	$e = 1; k = 8; m = 7; t = 4$	**28**	22	**0.850**	71.957	0.991
	DAUMI	$\rho = 1$	28	22	**0.994**	51.657	0.995
	DAUMI	$\underline{\rho = 9}$	27	22	0.974	**39.656**	0.996
	DAUMI	$\rho = 10$	26	22	0.972	42.321	0.996
	DAUMI	$\rho = 20$	24	22	0.968	41.700	0.996
	DAUMI	$\rho = 40$	**19**	22	0.954	40.810	0.996
Simulation 2	UMI-tools	$\underline{d = 1}$	21	23	**0.963**	**32.484**	0.997
	UMI-tools	$d = 2$	**18**	23	0.935	35.550	0.997
	Calib	$e = 1; k = 8; m = 5; t = 2$	23	23	0.884	71.070	0.993
	Calib	$e = 1; k = 8; m = 6; t = 2$	23	23	0.884	71.070	0.993
	Calib	$e = 1; k = 8; m = 6; t = 3$	23	23	0.884	71.070	0.993
	Calib	$\underline{e = 1; k = 8; m = 7; t = 2}$	23	23	0.884	71.070	0.993
	Calib	$e = 1; k = 8; m = 7; t = 3$	23	23	0.884	71.070	0.993
	Calib	$e = 1; k = 8; m = 7; t = 4$	**22**	23	**0.890**	**69.890**	**0.994**
	DAUMI	$\rho = 1$	28	23	1.015	76.834	0.995
	DAUMI	$\rho = 10$	28	23	1.015	33.834	0.998
	DAUMI	$\rho = 20$	27	23	1.010	34.503	0.998
	DAUMI	$\rho = 40$	17	23	0.995	23.691	0.998
	DAUMI	$\underline{\rho = 45}$	**16**	23	**0.995**	**22.691**	0.998
Simulation 3	UMI-tools	$\underline{d = 1}$	44	22	**0.549**	135.946	**0.967**
	UMI-tools	$d = 2$	**42**	22	0.477	**128.747**	0.959
	Calib	$e = 1; k = 8; m = 5; t = 2$	29	22	0.680	186.068	0.970
	Calib	$e = 1; k = 8; m = 6; t = 2$	31	22	0.658	187.988	0.968
	Calib	$e = 1; k = 8; m = 6; t = 3$	30	22	0.673	**184.127**	**0.970**
	Calib	$\underline{e = 1; k = 8; m = 7; t = 2}$	**28**	22	0.694	327.521	0.951
	Calib	$e = 1; k = 8; m = 7; t = 3$	**28**	22	0.694	327.521	0.951
	Calib	$e = 1; k = 8; m = 7; t = 4$	29	22	**0.698**	324.900	0.951
	DAUMI	$\rho = 1$	21	22	**0.975**	61.111	0.995
	DAUMI	$\rho = 10$	21	22	0.971	39.291	0.997
	DAUMI	$\rho = 20$	20	22	0.968	43.559	0.997
	DAUMI	$\rho = 40$	13	22	0.947	29.233	**0.998**
	DAUMI	$\underline{\rho = 49}$	**10**	22	0.932	**29.173**	0.997
Simulation 4	UMI-tools	$\underline{d = 1}$	44	21	**0.651**	**120.385**	**0.964**
	UMI-tools	$d = 2$	**39**	21	0.463	134.589	0.947
	Calib	$e = 1; k = 8; m = 5; t = 2$	42	22	0.661	253.790	0.950
	Calib	$e = 1; k = 8; m = 6; t = 2$	41	22	0.643	269.999	0.945
	Calib	$e = 1; k = 8; m = 6; t = 3$	**40**	22	**0.665**	**243.061**	**0.953**
	Calib	$\underline{e = 1; k = 8; m = 7; t = 2}$	41	22	0.651	303.096	0.940
	Calib	$e = 1; k = 8; m = 7; t = 3$	41	22	0.651	303.096	0.940
	Calib	$e = 1; k = 8; m = 7; t = 4$	42	22	0.654	301.254	0.940
	DAUMI	$\rho = 1$	31	22	**0.972**	**53.410**	**0.995**
	DAUMI	$\rho = 10$	31	22	0.936	69.267	0.993
	DAUMI	$\underline{\rho = 18}$	25	22	0.880	96.708	0.989
	DAUMI	$\rho = 20$	25	22	0.867	110.151	0.987
	DAUMI	$\rho = 40$	**19**	22	0.734	83.868	0.986

Table S7: Performance with known haplotypes $\mathcal{H}$ and varying penalty parameter ρ in simulation. Fitting the linear model, Deduplicated Abundance = $b\times$(True Abundance), we report R^2: proportion of variance explained; RSS: residual sum of squares; b: coefficient of true abundance; FP: Number of false positives (there are no false negatives); TP: Number of true positives; Dist.: centered scaled distance between ρ and expected amplified abundance, $(\rho - \mu)/s$. The theoretical mean (μ) and variance (s^2) and auto-selected ρ are reported in Table S2. Comparable results for $\mathcal{H}$ unknown in Figure 3 and Tables S5–S6. The best performer for each dataset is bolded unless there is a tie of more than two values of ρ.

Simulation	ρ	Dist.	FP	TP	b	RSS	R^2
1	0.01	-1.74	0	22	**0.999**	14.980	0.9987
	40	-0.53	0	22	0.988	**7.277**	**0.9993**
2	0.01	-4.38	0	23	1.016	4.426	0.9997
	40	-2.42	0	23	**1.015**	**2.834**	**0.9998**
3	0.01	-4.38	2	22	0.971	21.770	0.9982
	10	-3.89	0	22	**0.971**	16.770	0.9986
	20	-3.40	0	22	0.969	16.443	**0.9987**
	30	-2.91	0	22	0.966	16.976	0.9986
	40	-2.42	0	22	0.963	**15.214**	**0.9987**
	50	-1.93	0	22	0.946	33.594	0.9971
4	0.01	-1.74	1	22	**0.972**	**15.466**	**0.9985**
	10	-1.44	0	22	0.943	32.181	0.9968
	20	-1.13	0	22	0.887	76.858	0.9913
	30	-0.83	0	22	0.830	74.992	0.9903
	40	-0.53	0	22	0.772	79.438	0.9881
	50	-0.23	0	22	0.738	101.062	0.9836

Table S8: Information on the tested HIV amplicon datasets. Strand: forward or reverse strand used in the analysis; UMI Len. (nt): UMI length in nucleotides; Read Len. (nt): length of sampled sequence in nucleotides; The length of whole read is UMI Len. + Read Len.. No. reads: total number of reads in dataset.

**Dataset**	**Accession**	**Region**	**Strand**	**UMI Len. (nt)**	**Read Len. (nt)**	**No. reads**
V1V2	SRR2241783	V1V2	Reverse	9	241	33.5k
V3	SRR5105420	V3	Reverse	10	249	530.6k

Table S9: Agreement of DAUMI results on five random halvings of the V1V2 and V3 datasets as a function of ρ. Columns are as described for Table 1. The chosen ρ and the best performance for each column are bolded.

		**Deduplicated abundance $\geq$ 1**							**Deduplicated abundance $\geq$ 2**						
**Dataset**	ρ	**Hap1**	**Hap2**	**S.D.**	**Jaccard**		**Ruzicka**		**Hap1**	**Hap2**	**S.D.**	**Jaccard**		**Ruzicka**	
V1V2	1	90	91	(4)	0.70	(0.03)	0.81	(0.02)	46	47	(3)	0.64	(0.04)	0.81	(0.02)
	**10**	90	90	(5)	0.72	(0.03)	**0.85**	(0.01)	36	37	(2)	0.77	(0.04)	**0.87**	(0.01)
	20	88	89	(4)	0.73	(0.04)	0.84	(0.01)	36	37	(2)	0.76	(0.03)	0.86	(0.01)
	40	87	88	(4)	**0.73**	(0.04)	0.84	(0.01)	36	36	(2)	**0.77**	(0.03)	0.86	(0.01)
V3	1	183	174	(7)	0.39	(0.01)	**0.83**	(0.01)	96	96	(3)	0.74	(0.04)	**0.90**	(0.01)
	**10**	180	171	(7)	0.39	(0.01)	0.72	(0.01)	68	72	(4)	0.72	(0.03)	0.83	(0.01)
	20	179	171	(7)	0.40	(0.01)	0.68	(0.02)	63	65	(3)	0.73	(0.03)	0.74	(0.02)
	40	171	164	(7)	**0.40**	(0.01)	0.65	(0.01)	58	59	(3)	**0.75**	(0.05)	0.81	(0.01)

Table S10: Agreement of Calib, UMItools, Starcode-umi results on five random halvings of V1V2 and V3 datasets after removing singleton UMIs. Columns are as described for Table 1.

(a) Mean (standard deviation)

		**Deduplicated abundance $\geq$ 1**							**Deduplicated abundance $\geq$ 2**						
**Dataset**	**Methods**	**Hap1**	**Hap2**	**S.D.**	**Jaccard**		**Ruzicka**		**Hap1**	**Hap2**	**S.D.**	**Jaccard**		**Ruzicka**	
V1V2	Calib	183	180	(5)	0.45	(0.02)	0.69	(0.01)	37	38	(4)	0.76	(0.04)	0.86	(0.03)
	DAUMI	90	90	(5)	0.72	(0.03)	**0.84**	(0.02)	37	37	(2)	0.77	(0.05)	0.87	(0.02)
	Naïve	293	303	(9)	0.29	(0.01)	0.57	(0.01)	43	43	(3)	0.75	(0.03)	0.88	(0.03)
	Starcode-umi	16	14	(2)	**0.75**	(0.05)	0.81	(0.02)	11	10	(1)	0.71	(0.05)	0.81	(0.02)
	UMI-tools	187	199	(3)	0.50	(0.02)	0.74	(0.01)	39	40	(2)	**0.83**	(0.05)	**0.90**	(0.01)
V3	Calib	1091	1094	(25)	0.05	(0.00)	0.08	(0.00)	25	21	(2)	0.40	(0.04)	0.54	(0.02)
	DAUMI	180	171	(7)	**0.39**	(0.01)	**0.72**	(0.01)	68	72	(4)	**0.72**	(0.03)	**0.83**	(0.01)
	Naïve	1561	1543	(10)	0.06	(0.00)	0.22	(0.00)	91	84	(4)	0.64	(0.04)	0.74	(0.02)
	Starcode-umi	329	321	(13)	0.06	(0.00)	0.50	(0.00)	26	26	(4)	0.14	(0.02)	**0.83**	(0.01)
	UMI-tools	1308	1324	(18)	0.05	(0.00)	0.14	(0.00)	53	51	(4)	0.70	(0.04)	0.75	(0.03)

(b) Change in mean from Table 1

		**Deduplicated abundance $\geq$ 1**				**Deduplicated abundance $\geq$ 2**			
**Dataset**	**Method**	**ΔHap1**	**ΔHap2**	**ΔJaccard**	**ΔRuzicka**	**ΔHap1**	**ΔHap2**	**ΔJaccard**	**ΔRuzicka**
V1V2	Calib	-625	-617	0.37	0.46	-6	-5	0.09	0.04
	DAUMI								
	Naïve								
	Starcode-umi	-2288	-2297	0.75	0.62	-4	-3	-0.02	0.01
	UMI-tools	-355	-339	0.36	0.38	-3	-2	0.06	0.03
V3	Calib	-3957	-3927	0.05	-0.03	-39	-45	-0.15	-0.17
	DAUMI								
	Naïve								
	Starcode-umi	-14748	-14776	0.06	0.41	-70	-77	0.06	0.06
	UMI-tools	-2627	-2400	0.05	0.00	-19	-23	0.06	0.00

Table S11: DAUMI default settings are conservative for spike data. We report performance of DAUMI, UMI-tools and Naïve method on one single cell (cell barcode: TCGTAGACCAGATTCG) from the single-cell molecular spike dataset. DAUMI (AIC) shows results of DAUMI with a larger initial UMI set $\mathcal{U}$, prepared by AmpliCI with option `--useAIC`, which more liberally accepts haplotypes than the default BIC. Columns are as described for Figure 5 in the main text.

**Method**	**Precision**	**Recall**	**Jaccard**	**V-measure**	**Homogeneity**	**Completeness**
DAUMI (AIC)	0.898	0.892	0.810	0.965	0.933	0.999
DAUMI	0.913	0.791	0.736	0.957	0.919	0.997
UMI-tools	0.723	0.778	0.600	0.951	0.913	0.992
Naïve	0.810	0.972	0.793	0.967	0.941	0.994

References

Ameijeiras-Alonso, J. *et al.* (2021). multimode: An R package for mode assessment. *Journal of Statistical Software*, **97**(9), 1–32.

Amir, A. *et al.* (2017). Deblur rapidly resolves single-nucleotide community sequence patterns. *mSystems*, **2**(2), e00191–16.

Armagan, A. *et al.* (2013). Generalized double Pareto shrinkage. *Statistica Sinica*, **23**(1), 119–143.

Bhiman, J. N. *et al.* (2015). Viral variants that initiate and drive maturation of V1V2-directed HIV-1 broadly neutralizing antibodies. *Nature Medicine*, **21**(11), 1332–1336.

Blundell, J. R. and Levy, S. F. (2014). Beyond genome sequencing: Lineage tracking with barcodes to study the dynamics of evolution, infection, and cancer. *Genomics*, **104**(6, Part A), 417–430.

Callahan, B. J. *et al.* (2016). DADA2: High-resolution sample inference from Illumina amplicon data. *Nature Methods*, **13**(7), 581–583.

Callahan, B. J. *et al.* (2017). Exact sequence variants should replace operational taxonomic units in marker-gene data analysis. *The ISME Journal*, **11**(12), 2639–2643.

Candès, E. J. *et al.* (2008). Enhancing sparsity by reweighted ℓ_1 minimization. *Journal of Fourier Analysis and Applications*, **14**(5-6), 877–905.

Caskey, M. *et al.* (2017). Antibody 10-1074 suppresses viremia in HIV-1-infected individuals. *Nature Medicine*, **23**(2), 185–191.

Chen, H. and Boutros, P. C. (2011). VennDiagram: A package for the generation of highly-customizable Venn and Euler diagrams in R. *BMC Bioinformatics*, **12**(1), 35.

Chen, S. *et al.* (2019). Gencore: an efficient tool to generate consensus reads for error suppressing and duplicate removing of NGS data. *BMC Bioinformatics*, **20**(23), 606.

Clement, K. *et al.* (2018). AmpUMI: Design and analysis of unique molecular identifiers for deep amplicon sequencing. *Bioinformatics*, **34**(13), i202–i210.

Conway, J. R. *et al.* (2017). UpSetR: An R package for the visualization of intersecting sets and their properties. *Bioinformatics*, **33**(18), 2938–2940.

de Leeuw, J. (1994). Block-relaxation algorithms in statistics. In H.-H. Bock, W. Lenski, and M. M. Richter, editors, *Information Systems and Data Analysis*, pages 308–324. Springer Berlin Heidelberg, Berlin, Heidelberg.

DePristo, M. A. *et al.* (2011). A framework for variation discovery and genotyping using next-generation DNA sequencing data. *Nature Genetics*, **43**(5), 491–498.

Deza, E. and Deza, M.-M. (2006). *Dictionary of Distances*. Elsevier Science.

Dorman, K. S. *et al.* (2021). Denoising methods for inferring microbiome community content and abundance. In S. Datta and S. Guha, editors, *Statistical Analysis of Microbiome Data*, pages 3–25. Springer Nature, Cham, Switzerland.

Edgar, R. C. (2016). UNOISE2: Improved error-correction for Illumina 16S and ITS amplicon sequencing. *bioRxiv*.

Ewing, B. and Green, P. (1998). Base-calling of automated sequencer traces using Phred. II. error probabilities. *Genome Research*, **8**(3), 186–194.

Faust, G. G. and Hall, I. M. (2014). SAMBLASTER: Fast duplicate marking and structural variant read extraction. *Bioinformatics*, **30**(17), 2503–2505.

Fields, B. *et al.* (2021). MAUI-seq: Metabarcoding using amplicons with unique molecular identifiers to improve error correction. *Molecular Ecology Resources*, **21**(3), 703–720.

Galanti, L. *et al.* (2021). Pheniqs 2.0: accurate, high-performance bayesian decoding and confidence estimation for combinatorial barcode indexing. *BMC Bioinformatics*, **22**(1), 359.

Hagemann-Jensen, M. *et al.* (2020). Single-cell RNA counting at allele and isoform resolution using Smart-seq3. *Nature Biotechnology*, **38**(6), 708–714.

Hathaway, N. J. *et al.* (2018). SeekDeep: Single-base resolution de novo clustering for amplicon deep sequencing. *Nucleic Acids Research*, **46**(4), e21–e21.

Huang, W. *et al.* (2012). ART: A next-generation sequencing read simulator. *Bioinformatics*, **28**(4), 593–594.

Hubert, L. and Arabie, P. (1985). Comparing partitions. *Journal of Classification*, **2**, 193–218.

Hug, H. and Schuler, R. (2003). Measurement of the number of molecules of a single mRNA species in a complex mRNA preparation. *Journal of Theoretical Biology*, **221**(4), 615–624.

Jabara, C. B. *et al.* (2011). Accurate sampling and deep sequencing of the HIV-1 protease gene using a primer ID. *Proceedings of the National Academy of Sciences*, **108**(50), 20166–20171.

Jaccard, P. (1912). The distribution of the flora in the alpine zone. *New Phytologist*, **11**(2), 37–50.

Karst, S. M. *et al.* (2021). High-accuracy long-read amplicon sequences using unique molecular identifiers with Nanopore or PacBio sequencing. *Nature Methods*, **18**(2), 165–169.

Kebschull, J. M. and Zador, A. M. (2015). Sources of PCR-induced distortions in high-throughput sequencing data sets. *Nucleic Acids Research*, **43**(21), e143–e143.

Kebschull, J. M. and Zador, A. M. (2018). Cellular barcoding: Lineage tracing, screening and beyond. *Nature Methods*, **15**(11), 871–879.

Kim, T. H. *et al.* (2020). Demystifying "drop-outs" in single-cell UMI data. *Genome Biology*, **21**(1), 196.

Kinde, I. *et al.* (2011). Detection and quantification of rare mutations with massively parallel sequencing. *Proceedings of the National Academy of Sciences*, **108**(23), 9530–9535.

Kivioja, T. *et al.* (2012). Counting absolute numbers of molecules using unique molecular identifiers. *Nature Methods*, **9**(1), 72–74.

Klepikova, A. V. *et al.* (2017). Effect of method of deduplication on estimation of differential gene expression using RNA-seq. *PeerJ*, **5**, e3091.

König, J. *et al.* (2010). iCLIP reveals the function of hnRNP particles in splicing at individual nucleotide resolution. *Nature Structural & Molecular Biology*, **17**(77), 909–915.

Lange, K. (2010). *The Finite Fourier Transform*, chapter 20, pages 395–312. Statistics and Computing. Springer, 2nd edition.

Li, H. *et al.* (2009). The sequence alignment/map format and SAMtools. *Bioinformatics*, **25**(16), 2078–2079.

McKenna, A. and Gagnon, J. A. (2019). Recording development with single cell dynamic lineage tracing. *Development*, **146**(12), dev169730.

McLachlan, G. J. and Krishnan, T. (2008). *The EM Algorithm and Extensions*. Wiley Series in Probability and Statistics. John Wiley & Sons, 2nd edition.

Miner, B. E. *et al.* (2004). Molecular barcodes detect redundancy and contamination in hairpin-bisulfite PCR. *Nucleic Acids Research*, **32**(17), e135–e135.

Newman, A. M. *et al.* (2016). Integrated digital error suppression for improved detection of circulating tumor DNA. *Nature Biotechnology*, **34**(5), 547–555.

Orabi, B. *et al.* (2018). Alignment-free clustering of UMI tagged DNA molecules. *Bioinformatics*, **35**(11), 1829–1836.

Patro, R. *et al.* (2017). Salmon provides fast and bias-aware quantification of transcript expression. *Nature methods*, **14**(4), 417–419.

Peng, X. and Dorman, K. (2020). AmpliCI: A high-resolution model-based approach for denoising Illumina amplicon data. *Bioinformatics*, **36**(21), 5151–5158.

Petukhov, V. *et al.* (2018). dropEst: Pipeline for accurate estimation of molecular counts in droplet-based single-cell RNA-seq experiments. *Genome Biology*, **19**(1), 78.

Pflug, F. G. and von Haeseler, A. (2018). TRUmiCount: Correctly counting absolute numbers of molecules using unique molecular identifiers. *Bioinformatics*, **34**(18), 3137–3144.

Pokhilko, A. *et al.* (2021). Targeted single-cell RNA sequencing of transcription factors enhances the identification of cell types and trajectories. *Genome Research*, **31**(6), 1069–1081.

Potapov, V. and Ong, J. L. (2017). Examining sources of error in PCR by single-molecule sequencing. *PLOS ONE*, **12**(1), e0169774.

Quince, C. *et al.* (2011). Removing noise from pyrosequenced amplicons. *BMC Bioinformatics*, **12**(1), 38.

Rohland, N. and Reich, D. (2012). Cost-effective, high-throughput DNA sequencing libraries for multiplexed target capture. *Genome Research*, **22**(5), 939–946.

Rosati, E. *et al.* (2017). Overview of methodologies for T-cell receptor repertoire analysis. *BMC Biotechnology*, **17**(1), 61.

Rosenberg, A. and Hirschberg, J. (2007). V-measure: A conditional entropy-based external cluster evaluation measure. In *Proceedings of the 2007 Joint Conference on Empirical Methods in Natural Language Processing and Computational Natural Language Learning (EMNLP-CoNLL)*, pages 410–420, Prague, Czech Republic. Association for Computational Linguistics.

Sater, V. *et al.* (2020). UMI-VarCal: A new umi-based variant caller that efficiently improves low-frequency variant detection in paired-end sequencing NGS libraries. *Bioinformatics*, **36**(9), 2718–2724.

Schirmer, M. *et al.* (2016). Illumina error profiles: Resolving fine-scale variation in metagenomic sequencing data. *BMC Bioinformatics*, **17**(1), 125.

Seifert, D. *et al.* (2016). A comprehensive analysis of primer IDs to study heterogeneous HIV-1 populations. *Journal of Molecular Biology*, **428**(1), 238–250.

Sena, J. A. *et al.* (2018). Unique molecular identifiers reveal a novel sequencing artefact with implications for RNA-seq based gene expression analysis. *Scientific Reports*, **8**(1), 13121.

Shu, Y. *et al.* (2017). Circulating tumor DNA mutation profiling by targeted next generation sequencing provides guidance for personalized treatments in multiple cancer types. *Scientific Reports*, **7**(1), 583.

Shugay, M. *et al.* (2014). Towards error-free profiling of immune repertoires. *Nature Methods*, **11**(6), 653–655.

Shugay, M. *et al.* (2017). MAGERI: Computational pipeline for molecular-barcoded targeted resequencing. *PLOS Computational Biology*, **13**(5), 1–17.

Smith, T. *et al.* (2017). UMI-tools: Modeling sequencing errors in unique molecular identifiers to improve quantification accuracy. *Genome Research*, **27**(3), 491–499.

Srivastava, A. *et al.* (2019). Alevin efficiently estimates accurate gene abundances from dscRNA-seq data. *Genome Biology*, **20**(1), 65.

Stephens, M. A. (1974). EDF statistics for goodness of fit and some comparisons. *Journal of the American Statistical Association*, **69**(347), 730–737.

Stern, J. N. H. *et al.* (2014). B cells populating the multiple sclerosis brain mature in the draining cervical lymph nodes. *Science Translational Medicine*, **6**(248), 248ra107–248ra107.

Stoler, N. and Nekrutenko, A. (2021). Sequencing error profiles of Illumina sequencing instruments. *NAR Genomics and Bioinformatics*, **3**(1), lqab019.

Stoler, N. *et al.* (2016). Streamlined analysis of duplex sequencing data with Du Novo. *Genome Biology*, **17**(1), 180.

Stolovitzky, G. and Cecchi, G. (1996). Efficiency of DNA replication in the polymerase chain reaction. *Proceedings of the National Academy of Sciences*, **93**(23), 12947–12952.

Svensson, V. (2020). Droplet scRNA-seq is not zero-inflated. *Nature Biotechnology*, **38**(2), 147–150.

Sze, M. A. and Schloss, P. D. (2019). The impact of DNA polymerase and number of rounds of amplification in PCR on 16S rRNA gene sequence data. *mSphere*, **4**(3), e00163–19.

Townes, F. W. *et al.* (2019). Feature selection and dimension reduction for single-cell RNA-seq based on a multinomial model. *Genome Biology*, **20**(1), 295.

Vander Heiden, J. A. *et al.* (2014). pRESTO: a toolkit for processing high-throughput sequencing raw reads of lymphocyte receptor repertoires. *Bioinformatics*, **30**(13), 1930–1932.

Varghese, V. *et al.* (2010). Nucleic acid template and the risk of a PCR-induced HIV-1 drug resistance mutation. *PLOS ONE*, **5**(6), 1–6.

Woyke, T. and Jarett, J. (2015). Function-driven single-cell genomics. *Microbial Biotechnology*, **8**(1), 38–39.

Xu, C. *et al.* (2018). smCounter2: an accurate low-frequency variant caller for targeted sequencing data with unique molecular identifiers. *Bioinformatics*, **35**(8), 1299–1309.

Yin, X. (2016). *Probabilistic Methods for Quality Improvement in High-Throughput Sequencing Data*. Ph.D. thesis, Iowa State University.

Zanini, F. *et al.* (2017). Error rates, PCR recombination, and sampling depth in HIV-1 whole genome deep sequencing. *Virus Research*, **239**, 106–114.

Zhang, P. *et al.* (2021). Rbec: a tool for analysis of amplicon sequencing data from synthetic microbial communities. *ISME Communications*, **1**(1), 73.

Zhou, S. *et al.* (2015). Primer ID validates template sampling depth and greatly reduces the error rate of next-generation sequencing of HIV-1 genomic RNA populations. *Journal of Virology*, **89**(16), 8540–8555.

Ziegenhain, C. *et al.* (2022). Molecular spikes: a gold standard for single-cell RNA counting. *Nature Methods*, **19**(5), 560–566.

Zilionis, R. *et al.* (2017). Single-cell barcoding and sequencing using droplet microfluidics. *Nature Protocols*, **12**(1), 44–73.

Zorita, E. *et al.* (2015). Starcode: Sequence clustering based on all-pairs search. *Bioinformatics*, **31**(12), 1913–1919.
